# Supplementary material for: Mitotic chromosome binding predicts transcription factor properties in interphase
Source: Nat Commun. 2019 Jan 30;10:487. doi: 10.1038/s41467-019-08417-5 (PMC6353955; doi:10.1038/s41467-019-08417-5)
Supplement: Supplementary file 1 — Supplementary Information [file 41467_2019_8417_MOESM1_ESM.pdf]

Mitotic chromosome binding predicts transcription factor properties in interphase

Raccaud et al.

Supplementary Information

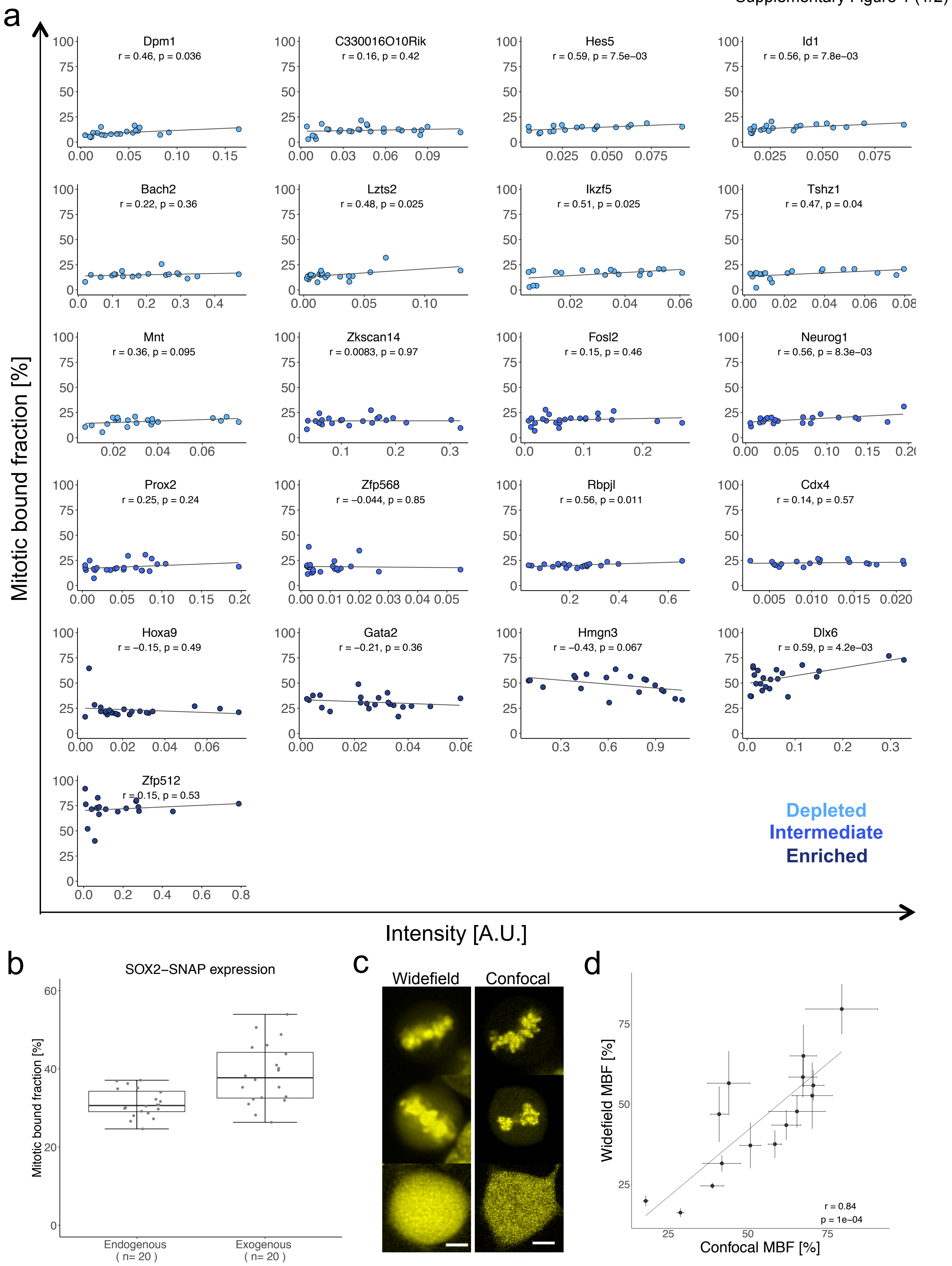
a
Mitotic bound fraction [%]
Intensity [A.U.]
Dpm1
r = 0.46, p = 0.036
C330016O10Rik
r = 0.16, p = 0.42
Hes5
r = 0.59, p = 7.5e−03
Id1
r = 0.56, p = 7.8e−03
Bach2
r = 0.22, p = 0.36
Lzts2
r = 0.48, p = 0.025
Ikzf5
r = 0.51, p = 0.025
Tshz1
r = 0.47, p = 0.04
Mnt
r = 0.36, p = 0.095
Zkscan14
r = 0.0083, p = 0.97
Fosl2
r = 0.15, p = 0.46
Neurog1
r = 0.56, p = 8.3e−03
Prox2
r = 0.25, p = 0.24
Zfp568
r = −0.044, p = 0.85
Rbpjl
r = 0.56, p = 0.011
Cdx4
r = 0.14, p = 0.57
Hoxa9
r = −0.15, p = 0.49
Gata2
r = −0.21, p = 0.36
Hmgn3
r = −0.43, p = 0.067
Dlx6
r = 0.59, p = 4.2e−03
Zfp512
r = 0.15, p = 0.53
Depleted
Intermediate
Enriched
b
SOX2−SNAP expression
Mitotic bound fraction [%]
Endogenous
(n= 20)
Exogenous
(n= 20)
c
Widefield
Confocal
d
Widefield MBF [%]
Confocal MBF [%]
r = 0.84
p = 1e−04

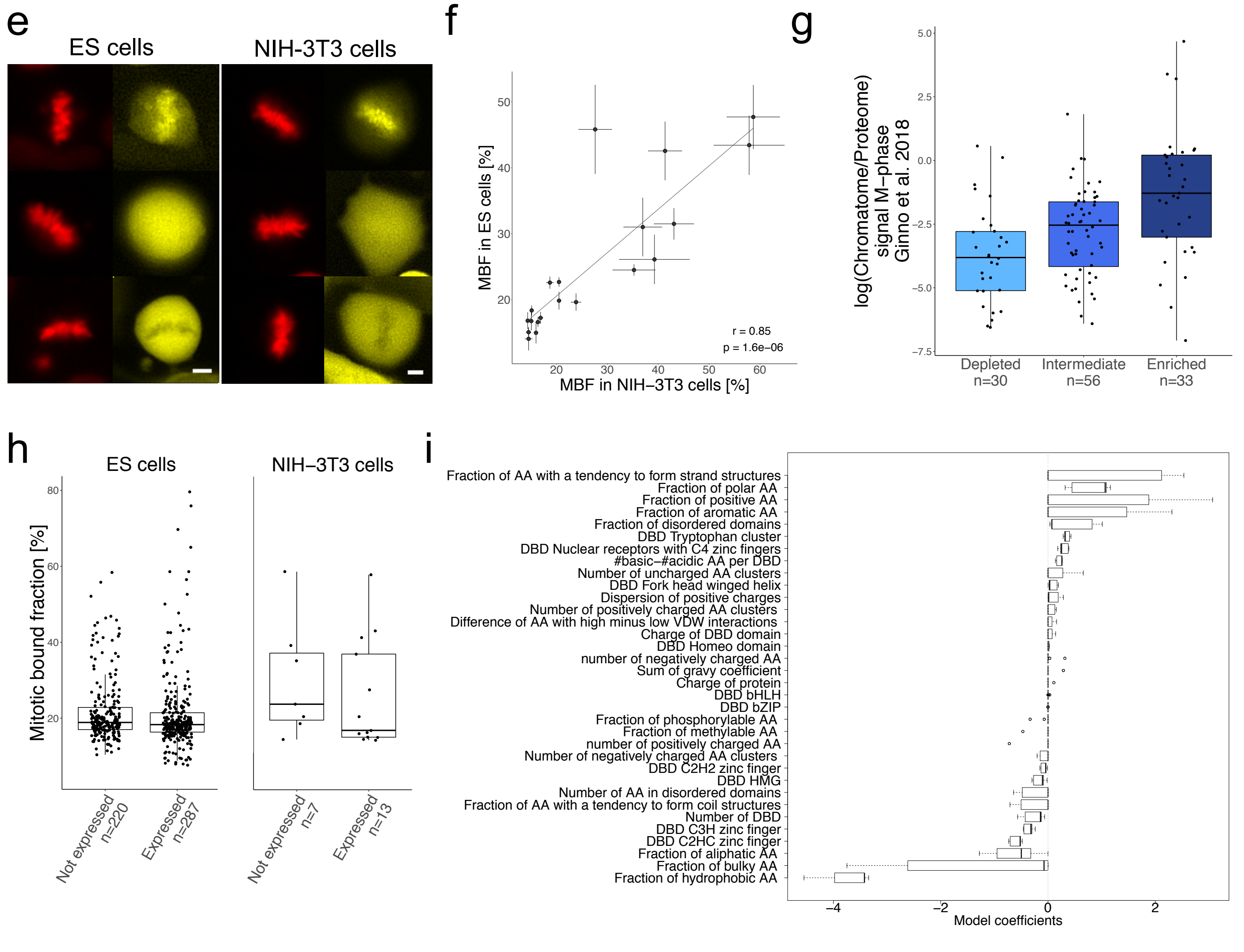

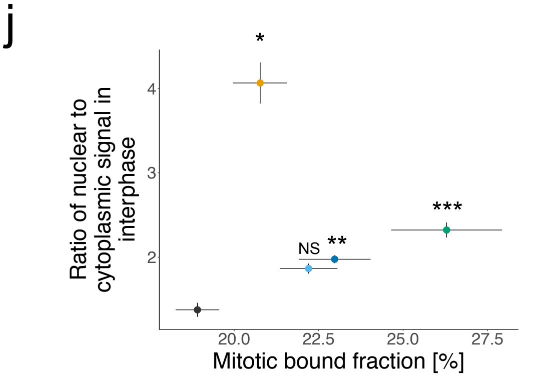

	Constructs	Added sequence	Positive charge added
● (orange)	YPet-NLS	RKRKR	5
● (green)	YPet-5xArginine	RRRRR	5
● (light blue)	YPet-NLS-1Insertion	RK**A**RKR	5
● (dark blue)	YPet-NLS-2Insertions	RK**A**RK**A**R	5
● (black)	YPet		0

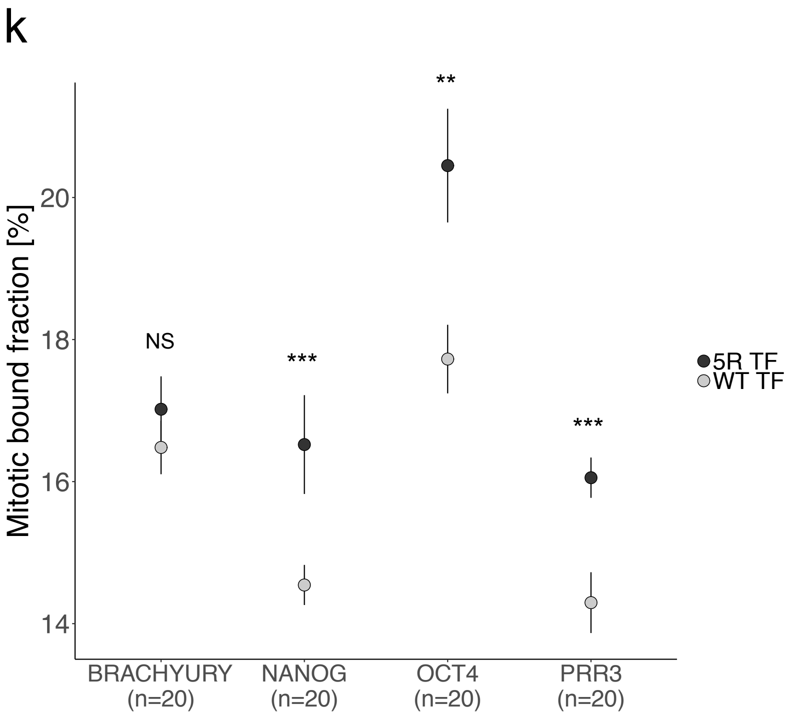

Supplementary Figure 1. Examples of MBF measurements by confocal microscopy in ES cells, MBF measurements in NIH-3T3 cells, additional parameters impacting the MBF, and impact of positively charged amino acids on the MBF. Related to Fig. 1.

a: Correlations between TF expression and mitotic bound fraction for all TFs with at least 19 cells quantified, sorted from lower to higher mitotic bound fraction.

b: Comparison of mitotic bound fractions for endogenous or exogenous (overexpression) of SOX2-SNAP.

c: Examples of TF-YPet localization in metaphase cells imaged with wide-field or confocal microscopy. Scale bar: 5µM.

d: Correlation between the mitotic bound fractions measured by wide-field or confocal microscopy (n=10). Error bars: SEM.

e: Examples of TF-YPet localization in metaphase cells imaged in ES cells and NIH-3T3 cells. Scale bar: 5µM.

f: Correlation between the mitotic bound fractions measured in ES cells and NIH-3T3 cells (for n values see Supplementary Table 3). Error bars: SEM.

g: Log ratio of Chromatome over Proteome signal of TFs in M-phase cells from Ginno et al. 2018 in the Depleted, Intermediate, and Enriched bins from this study.

h: MBFs of TFs expressed endogenously or not in ES cells and NIH-3T3 cells.

i: Quantification of the impact of all machine learning parameters that were correlated with the mitotic bound fraction and retained at least once by the algorithm (n=500 runs).

j: Impact of the NLS sequence or positively charged amino acids on the co-localization of YPet with mitotic chromosomes. (n>10) Error bars: SEM. $*$ $p < 0.05$; $**$ $p < 0.01$; $***$ $p < 0.001$; NS. not significant. P-values were obtained using a Wilcoxon rank-sum test.

k: Comparison of mitotic bound fraction of wildtype TFs (WT) or N-terminally tagged with five arginine residues (5R). Error bars: SEM. $**$ $p < 0.01$; $***$ $p < 0.001$; NS. not significant.

Boxplots: Boxes: intervals between the 25th and 75th percentile and median (horizontal line). Error bars: 1.5-fold the interquartile range or the closest data point when no data point is outside this range. P-values were obtained using a Wilcoxon rank-sum test.

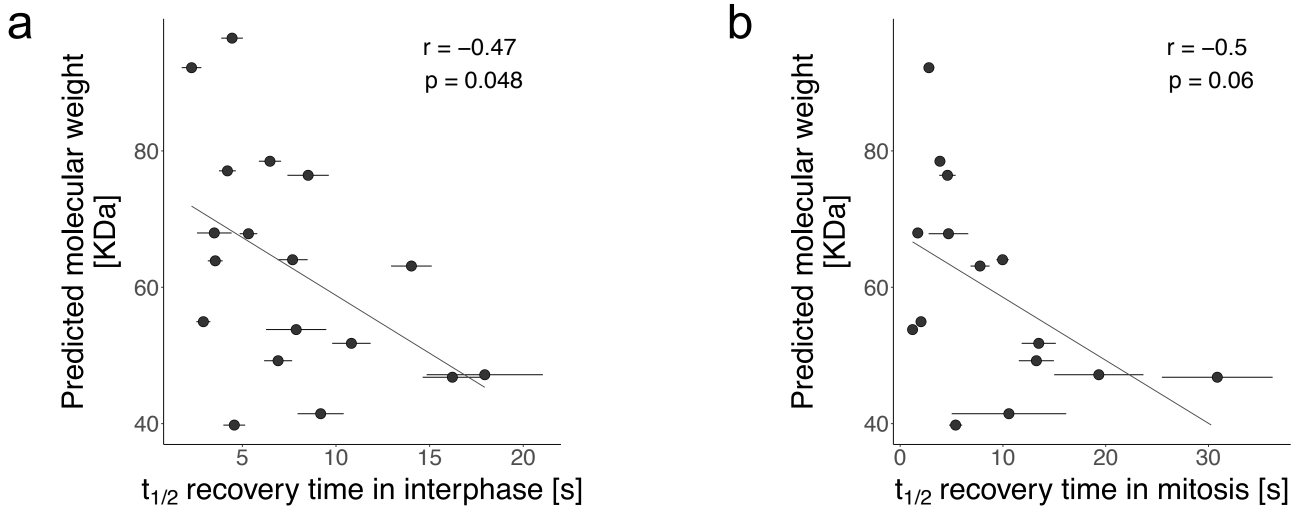
a
r = –0.47
p = 0.048
Predicted molecular weight [KDa]
80
60
40
5
10
15
20
t1/2 recovery time in interphase [s]
b
r = –0.5
p = 0.06
Predicted molecular weight [KDa]
80
60
40
0
10
20
30
t1/2 recovery time in mitosis [s]

Supplementary Figure 2: Correlation of TF mobility with TF size. Related to Fig. 3.

a: Correlation between FRAP $t_{1/2}$ recovery in interphase and the predicted molecular weight of transcription factors.
b: Correlation between FRAP $t_{1/2}$ recovery in mitosis and the predicted molecular weight of transcription factors.
Error bars: SEM (n=10).

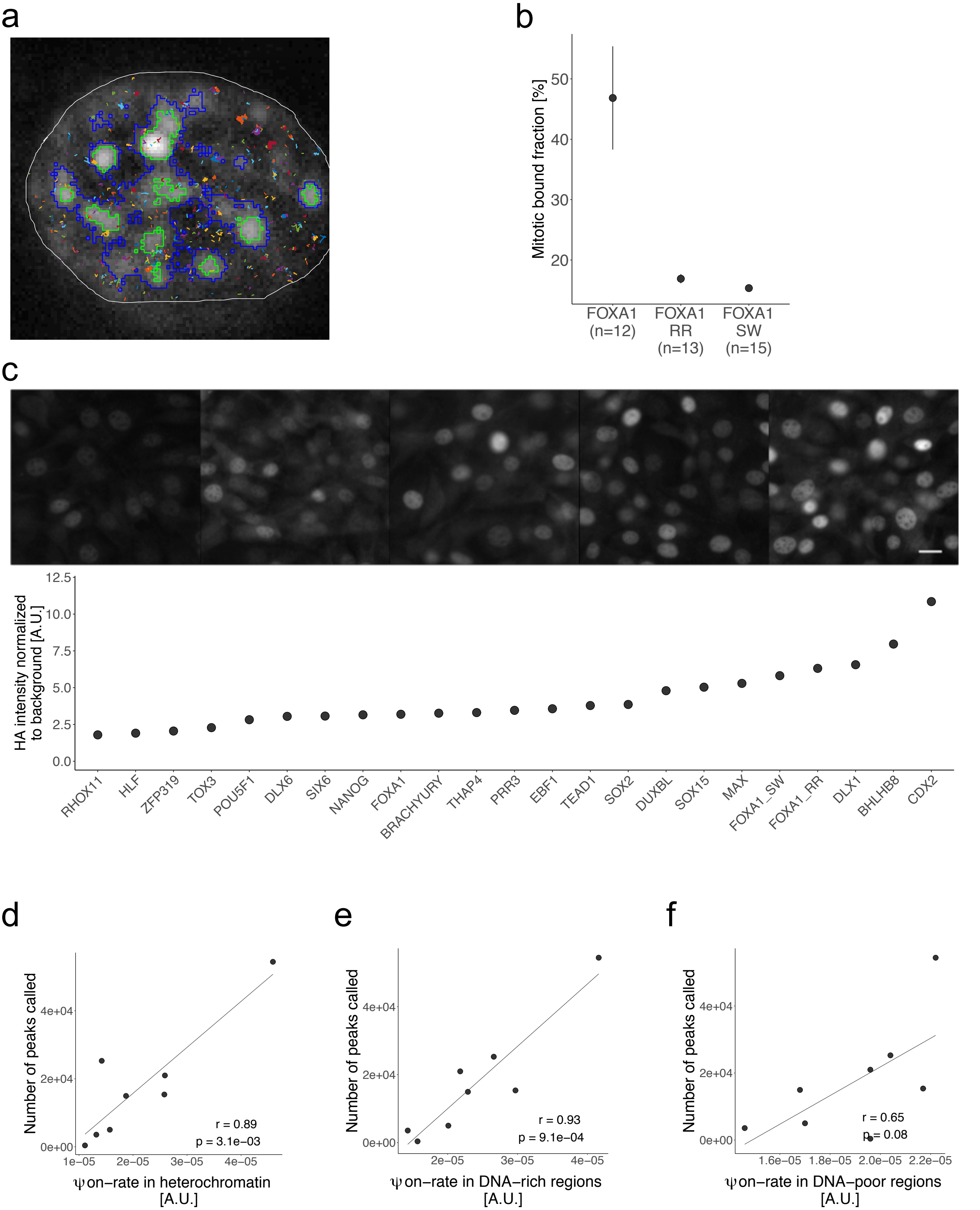
a
b
Mitotic bound fraction [%]
50
40
30
20
FOXA1 (n=12)
FOXA1 RR (n=13)
FOXA1 SW (n=15)
c
HA intensity normalized to background [A.U.]
12.5
10.0
7.5
5.0
2.5
0.0
RHOX11
HLF
ZFP319
TOX3
POU5F1
DLX6
SIX6
NANOG
FOXA1
BRACHYURY
THAP4
PRR3
EBF1
TEAD1
SOX2
DUXBL
SOX15
MAX
FOXA1_SW
FOXA1_RR
DLX1
BHLHB8
CDX2
d
Number of peaks called
4e+04
2e+04
0e+00
r = 0.89
p = 3.1e−03
1e−05
2e−05
3e−05
4e−05
ψ on−rate in heterochromatin [A.U.]
e
Number of peaks called
4e+04
2e+04
0e+00
r = 0.93
p = 9.1e−04
2e−05
3e−05
4e−05
ψ on−rate in DNA−rich regions [A.U.]
f
Number of peaks called
4e+04
2e+04
0e+00
r = 0.65
p = 0.08
1.6e−05
1.8e−05
2.0e−05
2.2e−05
ψ on−rate in DNA−poor regions [A.U.]

Supplementary Figure 3: Single molecule imaging of TF-DNA interactions. Related to Fig. 4.

a: Representative image of single molecule imaging of TFs. Regions displaying different densities of DNA that we automatically detected are shown in green (heterochromatin) and blue (DNA-dense). Tracks of single molecule events are displayed as in all other colors.

b: Mitotic bound fraction of FOXA1, FOXA1_RR and FOXA1_SW mutants. Error bars: SEM.

c: Selected images of immunofluorescence staining (anti-HA.11 IgG, BioLegend, # 901501, 1:500) of the HA-tagged TFs in selected dox-inducible cell lines (top) and HA signal quantification for all TF-3xHAs (bottom). Scale bar: 20μm.

d-f: Correlation between the ψon-rate in heterochromatic (**d**), DNA-rich (**e**) and DNA-poor (**f**) regions and the number of ChIP-seq peaks called.

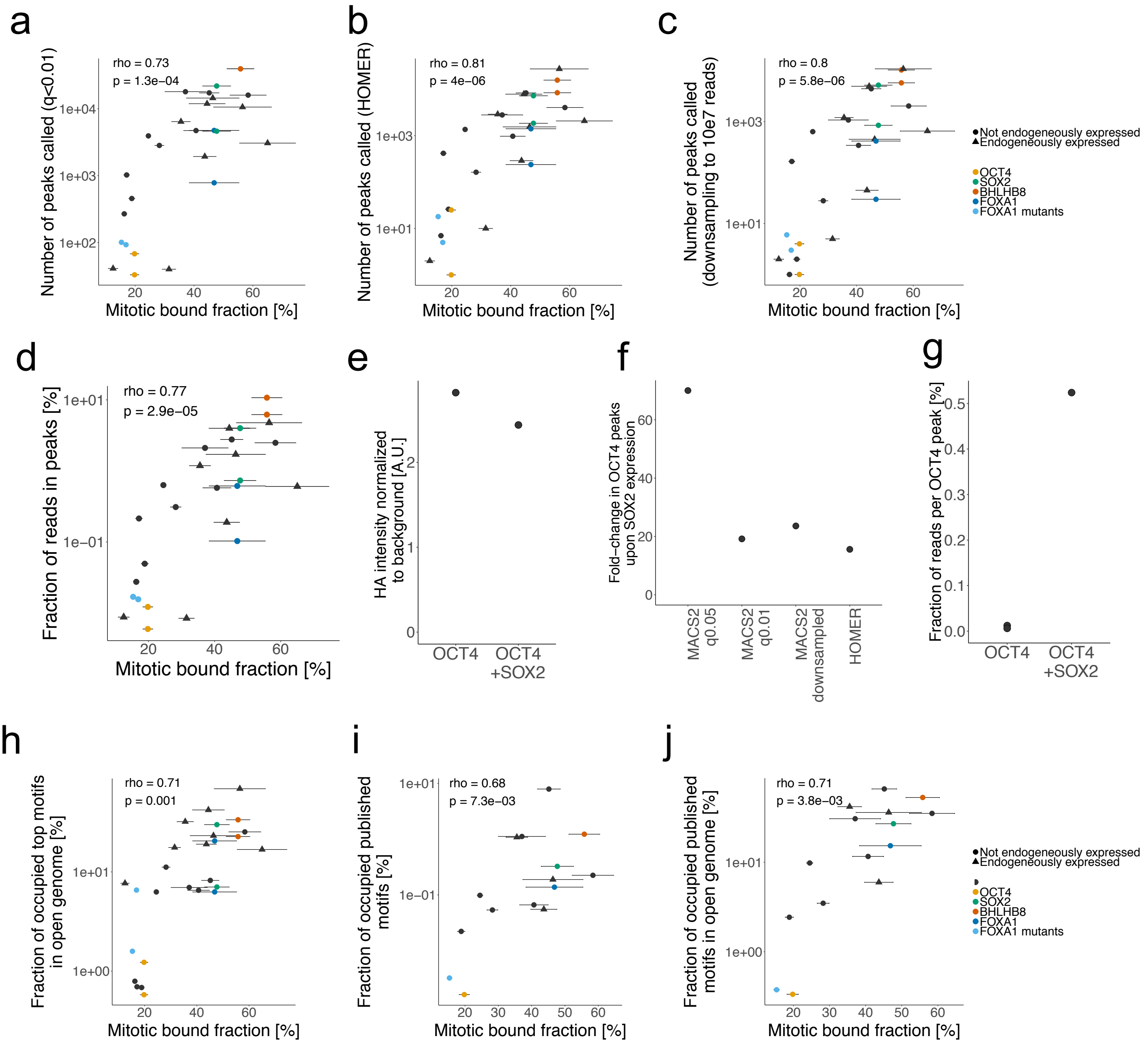
a
rho = 0.73
p = 1.3e-04
Number of peaks called (q<0.01)
Mitotic bound fraction [%]
b
rho = 0.81
p = 4e-06
Number of peaks called (HOMER)
Mitotic bound fraction [%]
c
rho = 0.8
p = 5.8e-06
Number of peaks called (downsampling to 10e7 reads)
Mitotic bound fraction [%]
Not endogeneously expressed
Endogeneously expressed
OCT4
SOX2
BHLHB8
FOXA1
FOXA1 mutants
d
rho = 0.77
p = 2.9e-05
Fraction of reads in peaks [%]
Mitotic bound fraction [%]
e
HA intensity normalized to background [A.U.]
OCT4
OCT4 +SOX2
f
Fold-change in OCT4 peaks upon SOX2 expression
MACS2 q0.05
MACS2 q0.01
MACS2 downsampled
HOMER
g
Fraction of reads per OCT4 peak [%]
OCT4
OCT4 +SOX2
h
rho = 0.71
p = 0.001
Fraction of occupied top motifs in open genome [%]
Mitotic bound fraction [%]
i
rho = 0.68
p = 7.3e-03
Fraction of occupied published motifs [%]
Mitotic bound fraction [%]
j
rho = 0.71
p = 3.8e-03
Fraction of occupied published motifs in open genome [%]
Mitotic bound fraction [%]
Not endogeneously expressed
Endogeneously expressed
OCT4
SOX2
BHLHB8
FOXA1
FOXA1 mutants

Supplementary Figure 4: Mitotic chromosome binding predicts genome-wide TF occupancy. Related to Fig. 4.

a: Correlation between the mitotic bound fraction and the number of ChIP-seq peaks called with MACS2 using a q-value cutoff of 0.01. Duplicates are indicated for OCT4 (yellow), SOX2 (green), BHLHB8 (red), and FOXA1 (dark blue). The two FOXA1 mutants are shown in light blue. Triangles: TFs endogenously expressed in NIH-3T3. Circles: TFs not endogenously expressed in NIH-3T3.

b: Correlation between the mitotic bound fraction and the number of ChIP-seq peaks called with HOMER. Same color- and shape-coding as Supplementary Fig. 4a.

c: Correlation between the mitotic bound fraction and the number of ChIP-seq peaks called with MACS2 using a downsampling to 10 million reads per samples. Same color- and shape-coding as Supplementary Fig. 4a.

d: Correlation between the mitotic bound fraction and the fraction of ChIP-seq reads in peaks (MACS2 $q<0.05$). Same color- and shape-coding as Supplementary Fig. 4a.

e: HA signal quantification for OCT4-3xHA in the presence of YPet-SOX2 in NIH-3T3 cells compared to OCT-3xHA alone.

f: Fold-change of the number of ChIP-seq peaks called for OCT4-3xHA in the presence of YPet-SOX2 compared to OCT-3xHA alone using different peak calling settings/algorithms.

g: Fraction of reads in peaks recovered for OCT4-3xHA in the presence of YPet-SOX2 in NIH-3T3 cells compared to OCT-3xHA alone (MACS2 $q<0.05$).

h: Correlation between the mitotic bound fraction and the number of peaks overlapping with the most frequently found motif for each TF in accessible chromatin, normalized over the total number of motif occurrence in the genomic regions encompassing accessible chromatin. Same color- and shape-coding as Supplementary Fig. 4a.

i: Correlation between the mitotic bound fraction and the number of peaks overlapping with the published TF motif, normalized over the total number of motif occurrences in the genome. Same color- and shape-coding as Supplementary Fig. 4a.

j: Correlation between the mitotic bound fraction and the number of peaks in accessible chromatin overlapping with the published TF motif, normalized over the total number of motif occurrences in the accessible genome. Same color- and shape-coding as Supplementary Fig. 4a.

a

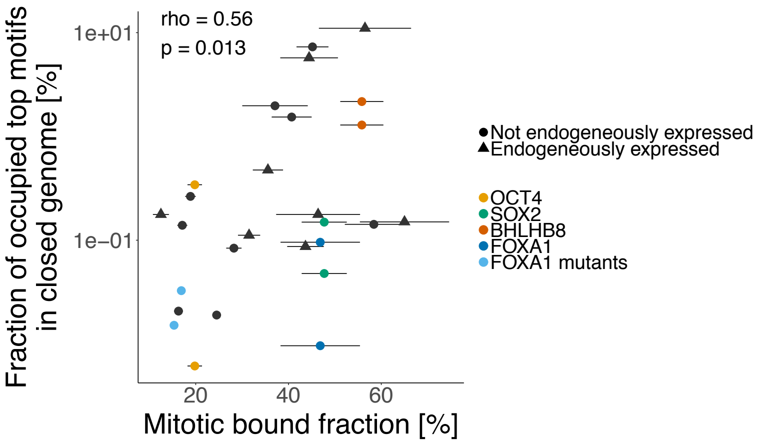
rho = 0.56
p = 0.013
1e+01
1e−01
Fraction of occupied top motifs in closed genome [%]
20
40
60
Mitotic bound fraction [%]
Not endogeneously expressed
Endogeneously expressed
OCT4
SOX2
BHLHB8
FOXA1
FOXA1 mutants

b

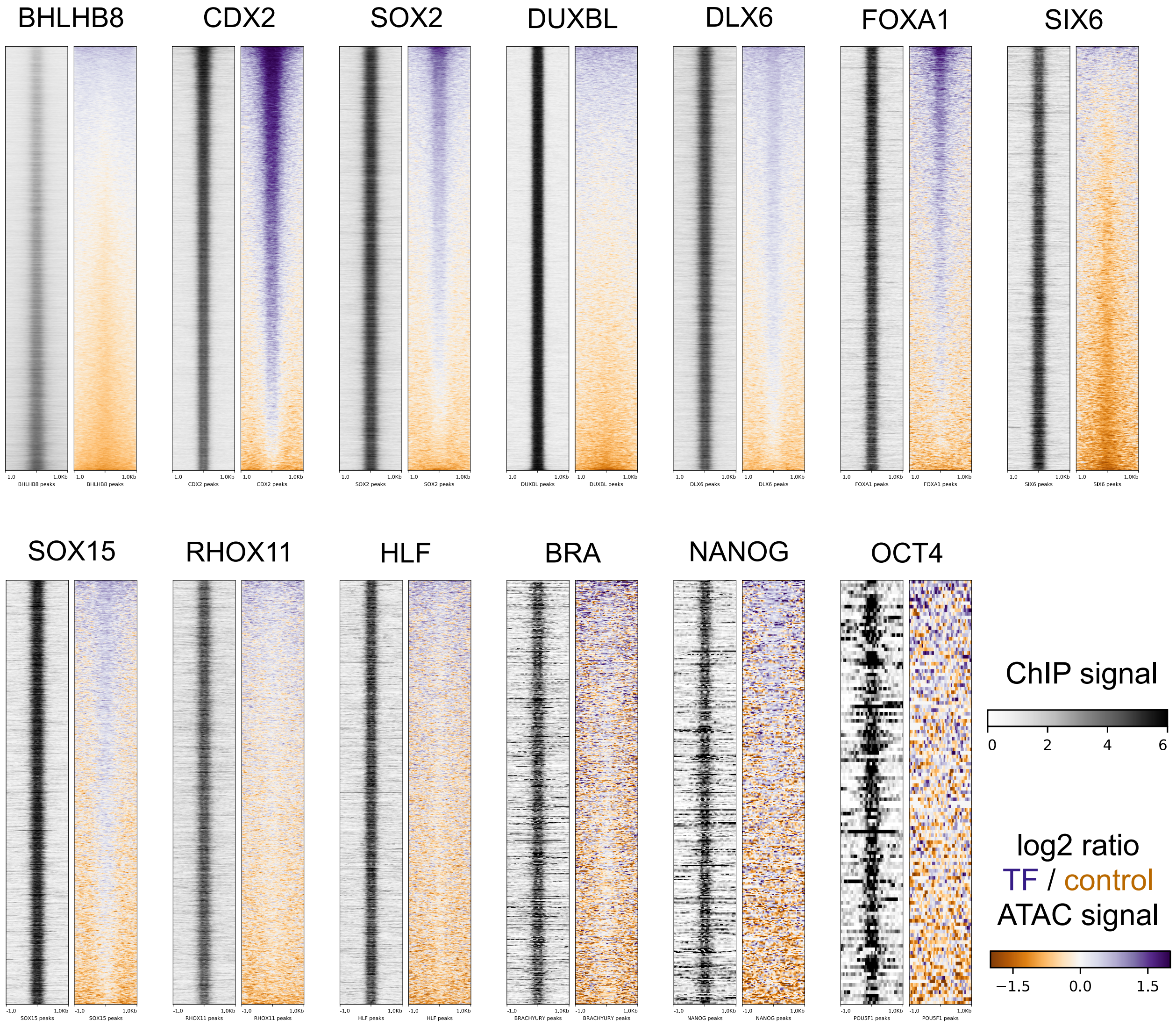
BHLHB8
CDX2
SOX2
DUXBL
DLX6
FOXA1
SIX6
SOX15
RHOX11
HLF
BRA
NANOG
OCT4
ChIP signal
0
2
4
6
log2 ratio
TF / control
ATAC signal
−1.5
0.0
1.5

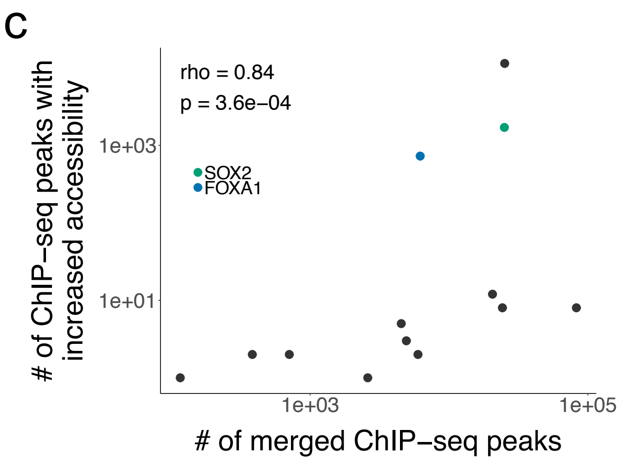
c
rho = 0.84
p = 3.6e−04
SOX2
FOXA1
1e+03
1e+01
# of ChIP−seq peaks with increased accessibility
1e+03
1e+05
# of merged ChIP−seq peaks

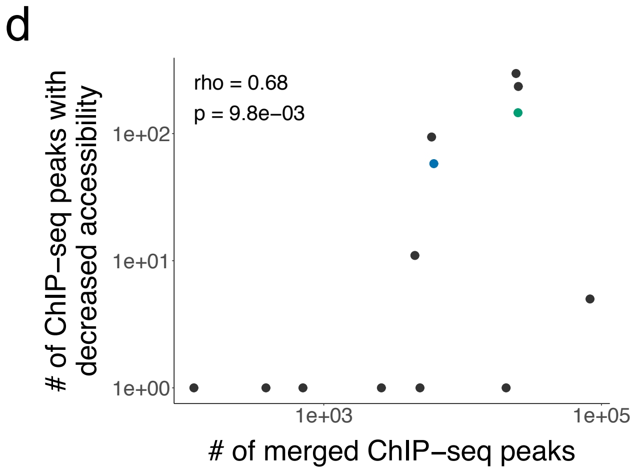
d
rho = 0.68
p = 9.8e−03
1e+02
1e+01
1e+00
# of ChIP−seq peaks with decreased accessibility
1e+03
1e+05
# of merged ChIP−seq peaks

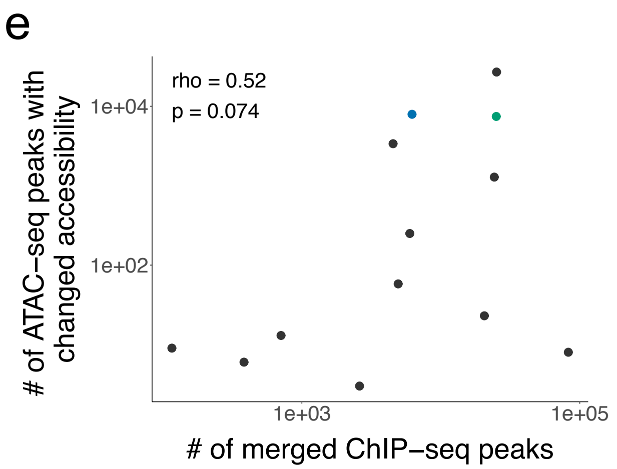
e
rho = 0.52
p = 0.074
1e+04
1e+02
# of ATAC−seq peaks with changed accessibility
1e+03
1e+05
# of merged ChIP−seq peaks

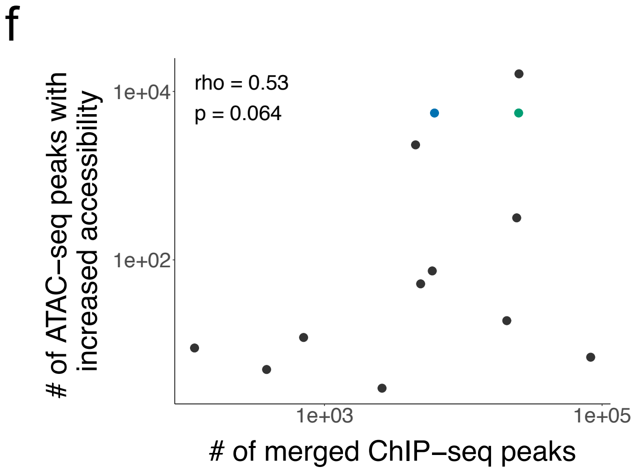
f
rho = 0.53
p = 0.064
1e+04
1e+02
# of ATAC−seq peaks with increased accessibility
1e+03
1e+05
# of merged ChIP−seq peaks

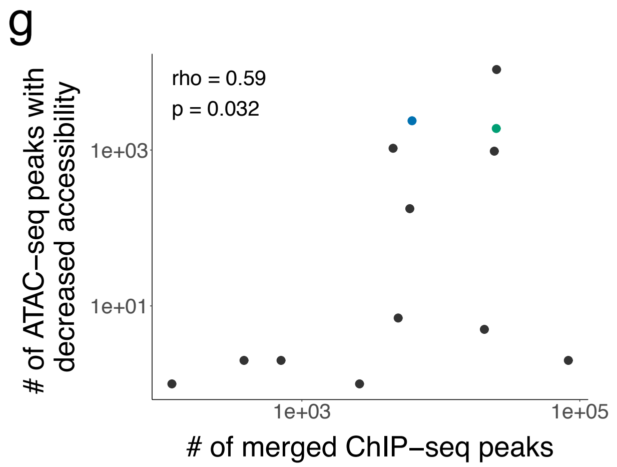
g
rho = 0.59
p = 0.032
1e+03
1e+01
# of ATAC−seq peaks with decreased accessibility
1e+03
1e+05
# of merged ChIP−seq peaks

Supplementary Figure 5: TF capacity to alter chromatin accessibility as a function of genome-wide occupancy and MBF. Related to Fig. 5.

a: Correlation between the mitotic bound fraction and the fraction of occupied top motifs in closed chromatin regions (devoid of ATAC-seq peak). Duplicates are indicated for OCT4 (yellow), SOX2 (green), BHLHB8 (red), and FOXA1 (dark blue). The two FOXA1 mutants are shown in light blue. Triangles: TFs endogenously expressed in NIH-3T3. Circles: TFs not endogenously expressed in NIH-3T3. Error bars: SEM.

b: Heatmaps showing ChIP-seq signal (left) and log2 ratio of ATAC-seq signal of TF overexpression over control (right) for all overexpressed factors 1 kb around the center of ChIP-seq peaks for each TF. Heatmaps are ordered vertically by log2 ratio for each factor and horizontally by number of ChIP-seq peaks per factor.

c: Correlation between the number of ChIP-seq peaks for each TF and the number of TF ChIP-seq peaks with significant increase of chromatin accessibility upon overexpression of the TF. Known pioneers TFs are indicated in green (SOX2) and blue (FOXA1).

d: Correlation between the number of ChIP-seq peaks for each TF and the number of TF ChIP-seq peaks with significant decrease of chromatin accessibility upon overexpression of the TF. Same color-coding as Supplementary Fig. 5c.

e: Correlation between the number of ChIP-seq peaks for each TF and the number of ATAC-seq peaks with significant change in chromatin accessibility upon overexpression of the TF. Same color-coding as Supplementary Fig. 5c.

f: Correlation between the number of ChIP-seq peaks for each TF and the number of ATAC-seq peaks with significant increase of chromatin accessibility upon overexpression of the TF. Same color-coding as Supplementary Fig. 5c.

g: Correlation between the number of ChIP-seq peaks for each TF and the number of ATAC-seq peaks with significant decrease of chromatin accessibility upon overexpression of the TF. Same color-coding as Supplementary Fig. 5c.

Supplementary Table 1. Mitotic bound fraction measured on confocal and wide-field microscopes. Related to Supplementary Fig. 1.

**Legend**	**Description**
**Transcription Factor**	Name of the transcription factor
**MBF confocal microscope**	Mitotic bound fraction measured by confocal microscopy
**SEM (MBF confocal microscope)**	Standard error of the mean for the mitotic bound fraction measured by confocal microscopy
**MBF widefield microscope**	Mitotic bound fraction measured by wide-field microscopy
**SEM (MBF widefield microscope)**	Standard error of the mean for the mitotic bound fraction measured by wide-field microscopy

**Transcription Factor**	**MBF confocal microscope [%]**	**SEM (MBF confocal microscope)**	**MBF wide-field microscope [%]**	**SEM (MBF wide-field microscope)**
Bhlhb8	70.5	3.4	55.8	4.6
Cdx2	50.7	3.3	37.1	7.1
Dlx6	67.2	4.3	58.4	6.2
Foxa1	40.9	2.6	46.8	8.6
Hmga2	70.2	6.3	52.6	10.2
Hmgb2	58.4	2.1	37.5	4.2
Hmgn1	62.0	4.5	43.4	4.5
Hoxd10	28.8	1.1	16.2	1.3
MafK	79.5	11.3	79.6	7.7
Max	67.4	4.1	65.0	9.6
Oct4	17.9	0.8	19.8	1.6
Sox15	38.8	3.6	24.5	0.9
Sox2	65.4	8.9	47.7	4.9
Tead1	44.0	6.6	56.5	9.9
Thap4	41.8	5.8	31.5	2.4

Supplementary Table 2. Mitotic bound fraction per transcription factor and associated colocalization group. Related to Figure 1.

Legend	Description
**Transcription Facto**	Name of the transcription factor
**MBF**	Mitotic bound fraction
**SEM(MBF)**	Standard error of the mean for the mitotic bound fraction
**n**	Number of ES cells used for MBF quantification
**Bin**	Group associated to the transcription factor as a function of the colocalization on mitotic chromosomes: Depleted, Intermediate, Enriched

Transcription Factor	MBF [%]	SEM(MBF)	n	Bin
Creb3l2	7.62	1.14	9	Depleted
Srebf2	7.91	0.87	10	Depleted
Zfp84	7.92	0.65	18	Depleted
Aire	8.13	2.01	10	Depleted
Tfam	8.33	0.89	14	Depleted
Zdhhc12	8.80	0.96	13	Depleted
Creb3	8.96	0.54	9	Depleted
Zdhhc3	8.99	0.76	10	Depleted
Zdhhc7	9.18	0.56	11	Depleted
Trmt1	9.35	1.24	12	Depleted
Zdhhc4	9.43	0.88	14	Depleted
Ikbkg	9.48	1.98	10	Depleted
Dpm1	9.50	0.80	21	Depleted
Zdhhc16	9.67	1.22	6	Depleted
Zdhhc19	10.36	1.05	12	Depleted
Zkscan6	10.58	1.73	13	Depleted
Dcun1d3	10.79	0.78	11	Depleted
Pias3	10.82	1.47	10	Depleted
Zdhhc20	11.01	0.87	13	Depleted
A830053O21Rik	11.04	0.99	15	Depleted
Zdhhc21	11.06	1.44	11	Depleted
Zdhhc1	11.41	1.02	11	Depleted
Lass2	11.67	0.52	10	Depleted
C330016O10Rik	11.90	0.91	28	Depleted
Mbnl3	11.97	1.09	10	Depleted
Nupl2	11.99	0.83	13	Depleted
Zdhhc9	12.02	0.92	13	Depleted
Prr3	12.50	1.72	11	Depleted
2410081M15Rik	12.70	1.53	11	Depleted
Mael	12.96	1.37	12	Depleted
Zdhhc25	13.10	1.60	14	Depleted
Lzts1	13.10	2.19	10	Depleted
Dzip1	13.29	1.47	10	Depleted
Zfp191	13.72	1.43	13	Depleted
Hes2	13.84	1.29	11	Depleted
Hes3	13.85	0.45	16	Depleted
Zdhhc14	13.97	1.94	13	Depleted
Sox13	13.97	1.38	11	Depleted
Nr0b1	14.07	1.77	14	Depleted
Hes5	14.09	0.75	19	Depleted
Zbtb20	14.09	0.89	17	Depleted
Zfp687	14.14	1.63	11	Depleted
Tbx10	14.16	2.82	10	Depleted
Lin28	14.23	1.09	17	Depleted
Tox	14.24	1.27	13	Depleted
Spert	14.26	1.05	11	Depleted
Hoxa13	14.26	1.15	18	Depleted
Mbnl2	14.29	1.09	11	Depleted
Rabgef1	14.30	2.69	10	Depleted
Gata5	14.38	2.20	11	Depleted
L3mbtl	14.40	1.18	18	Depleted
Tal2	14.45	1.59	12	Depleted
Phtf1	14.49	2.48	11	Depleted
Id1	14.55	0.81	21	Depleted
Olig3	14.61	1.22	5	Depleted
Zfp740	14.62	1.52	10	Depleted
Zfp282	14.79	1.17	10	Depleted
Mef2c	14.85	2.26	10	Depleted
Zc3hav1	14.85	1.76	13	Depleted
Rbm10	14.86	0.55	13	Depleted
Zbtb39	14.89	0.92	12	Depleted
6030490I01Rik	14.94	0.78	17	Depleted
Bcl6b	14.97	0.78	12	Depleted
Zfp661	14.99	1.28	14	Depleted
Zfp346	14.99	1.61	10	Depleted
AI894139	15.07	0.78	15	Depleted
Fam164a	15.08	0.63	18	Depleted
Mbnl1	15.16	1.36	10	Depleted
Bach2	15.17	0.98	19	Depleted
Lzts2	15.23	1.38	22	Depleted
Mkrn2	15.32	1.03	11	Depleted
Zfp365	15.32	0.69	11	Depleted
Prosapip1	15.37	0.53	15	Depleted
Foxp2	15.38	1.10	14	Depleted
Mef2b	15.39	0.74	12	Depleted
Zfp521	15.45	1.22	13	Depleted
Rnf113a2	15.46	1.53	14	Depleted
Smad2	15.47	1.46	10	Depleted
Stat1	15.50	1.10	10	Depleted
Rogdi	15.59	0.41	15	Depleted
Pbrm1	15.61	1.30	12	Depleted

Zfp446	15.62	1.38	11	Depleted
Dmrtc2	15.66	0.59	13	Depleted
Rela	15.71	1.33	13	Depleted
Mkrn1	15.73	2.52	15	Depleted
Stat3	15.82	1.07	13	Depleted
Ahr	15.83	0.82	10	Depleted
Arnt	15.84	0.95	17	Depleted
Hmbox1	15.89	1.35	12	Depleted
Tulp3	15.91	1.42	10	Depleted
6430604K15Rik	15.92	0.88	18	Depleted
Zfp92	15.95	0.74	10	Depleted
Pknox1	15.95	1.98	12	Depleted
Pbx1	15.99	0.90	12	Depleted
Ikzf5	16.01	1.40	19	Depleted
Zbtb12	16.02	1.26	10	Depleted
Zfp692	16.03	1.45	15	Depleted
Tshz1	16.03	1.20	19	Depleted
Mef2a	16.09	0.76	14	Depleted
Ascl3	16.14	1.02	10	Depleted
Arid3a	16.16	1.47	11	Depleted
Hoxd10	16.16	1.29	11	Depleted
Zfp647	16.16	2.03	11	Depleted
Gmeb1	16.17	1.54	11	Depleted
Zkscan1	16.20	0.89	10	Depleted
Ahrr	16.23	1.76	12	Depleted
Zfp108	16.24	0.25	11	Depleted
Tut1	16.26	1.09	12	Depleted
Mnt	16.28	0.95	22	Depleted
Foxm1	16.28	2.15	10	Depleted
Nanog	16.29	0.97	10	Depleted
Sall3	16.37	1.03	11	Depleted
Zfp57	16.38	2.40	10	Depleted
Spdef	16.40	0.90	11	Depleted
Klf1	16.44	0.98	11	Depleted
Klf4	16.45	1.56	13	Depleted
Tcf23	16.45	0.71	10	Depleted
Zfp536	16.47	0.75	10	Depleted
Lztr1	16.54	1.24	10	Intermediate
Lhx9	16.54	0.69	11	Intermediate
Zfp449	16.59	0.66	10	Intermediate
Fosl1	16.59	0.80	12	Intermediate
Rbm22	16.62	0.74	11	Intermediate
Rcor2	16.64	1.47	15	Intermediate
Vdr	16.70	1.11	13	Intermediate
Zfp414	16.77	1.40	11	Intermediate
Zbtb48	16.80	1.02	12	Intermediate
Npas4	16.82	1.28	11	Intermediate
Gm1008	16.82	0.97	10	Intermediate
A630042L21Rik	16.83	2.22	12	Intermediate
Pou6f2	16.90	0.53	10	Intermediate
Spic	16.95	0.71	12	Intermediate
Zgpat	16.95	1.13	10	Intermediate
Zkscan14	16.97	1.08	23	Intermediate
Rhox4a	16.97	1.01	17	Intermediate
Spz1	16.98	0.66	15	Intermediate
Gli1	16.99	1.10	10	Intermediate
Tbx20	16.99	0.53	10	Intermediate
Hnf1a	17.00	0.56	11	Intermediate
Zfp120	17.01	0.54	11	Intermediate
Csde1	17.02	0.58	11	Intermediate
Zfp639	17.03	0.77	12	Intermediate
Gmeb2	17.05	0.81	12	Intermediate
Zfp61	17.07	1.11	12	Intermediate
Zcchc4	17.08	0.86	10	Intermediate
Bcl11a	17.08	1.03	12	Intermediate
Ecd	17.10	0.57	13	Intermediate
Tox3	17.11	1.07	10	Intermediate
Zfp238	17.11	0.70	11	Intermediate
Rfx1	17.14	0.52	12	Intermediate
Nhlh1	17.15	0.83	16	Intermediate
Tcfeb	17.16	0.98	11	Intermediate
Meis2	17.19	0.62	13	Intermediate
Gbx2	17.20	0.88	12	Intermediate
BC023179	17.20	0.54	10	Intermediate
Dcun1d1	17.20	2.06	12	Intermediate
Klf2	17.21	1.07	14	Intermediate
Npas2	17.23	1.09	10	Intermediate
Pou6f1	17.23	2.06	12	Intermediate
Nufip1	17.23	1.59	14	Intermediate
Zfp809	17.25	0.89	10	Intermediate
Nr2c1	17.26	1.26	10	Intermediate
Fiz1	17.26	0.86	18	Intermediate
Neil3	17.26	0.76	11	Intermediate
Tead4	17.27	0.53	10	Intermediate
Hnf4g	17.31	0.58	10	Intermediate
Ctcf	17.31	1.80	15	Intermediate
2810426N06Rik	17.34	0.68	10	Intermediate
Erf	17.35	0.75	14	Intermediate
Arnt2	17.36	1.83	18	Intermediate
Bach1	17.36	0.60	9	Intermediate
Zfp513	17.38	0.58	15	Intermediate
Hoxb7	17.38	1.24	12	Intermediate

Chrac1	17.47	0.84	12	Intermediate
Fos	17.49	1.26	8	Intermediate
Foxp1	17.51	2.13	10	Intermediate
Arntl	17.51	1.42	11	Intermediate
Zfp7	17.52	1.18	8	Intermediate
Zfp219	17.54	1.49	17	Intermediate
Zfp704	17.54	1.50	12	Intermediate
Isx	17.55	0.67	12	Intermediate
Fezf2	17.55	1.14	9	Intermediate
Hesx1	17.60	0.68	8	Intermediate
Alx4	17.61	2.18	13	Intermediate
Zscan18	17.61	0.61	13	Intermediate
Nr4a1	17.62	0.72	13	Intermediate
BC043301	17.62	0.92	12	Intermediate
Hes7	17.66	1.03	14	Intermediate
Tbx19	17.67	0.54	11	Intermediate
Foxi2	17.67	1.51	14	Intermediate
Ddit3	17.68	0.60	14	Intermediate
Msgn1	17.70	2.33	16	Intermediate
Pax3	17.70	2.41	8	Intermediate
Zmat2	17.71	1.15	11	Intermediate
Rhox6	17.71	1.86	11	Intermediate
Sp3	17.71	1.57	10	Intermediate
Ep300	17.73	0.72	10	Intermediate
Zscan2	17.74	1.14	14	Intermediate
A830023I12Rik	17.76	1.09	13	Intermediate
Tbx15	17.78	0.90	14	Intermediate
Tbx22	17.78	2.16	10	Intermediate
Zfp12	17.80	0.72	11	Intermediate
Zfp358	17.81	1.49	11	Intermediate
Gtf3a	17.86	0.85	11	Intermediate
Adnp2	17.89	0.70	11	Intermediate
Egr1	17.89	1.15	9	Intermediate
Zfp85-rs1	17.92	0.98	10	Intermediate
Zfp46	17.93	0.88	11	Intermediate
Lhx3	17.93	1.47	13	Intermediate
Smarcc1	17.97	0.96	11	Intermediate
Usf1	18.00	1.76	12	Intermediate
Relb	18.01	0.89	13	Intermediate
Fosl2	18.05	1.16	25	Intermediate
Mycn	18.06	0.59	16	Intermediate
Jazf1	18.10	1.09	11	Intermediate
Bmyc	18.10	1.26	5	Intermediate
Zscan21	18.11	1.57	10	Intermediate
Klf5	18.12	1.09	10	Intermediate
Egr2	18.13	0.54	17	Intermediate
Hoxb5	18.14	0.67	14	Intermediate
Zfp14	18.16	0.81	12	Intermediate
Tsc22d3	18.16	1.40	14	Intermediate
tcf7	18.17	0.73	10	Intermediate
Esr2	18.18	1.25	11	Intermediate
Prrxl1	18.18	1.00	11	Intermediate
Batf2	18.19	1.10	10	Intermediate
Tcfcp2l1	18.22	0.86	13	Intermediate
Sall4	18.23	1.11	14	Intermediate
Churc1	18.28	0.44	11	Intermediate
Zfp689	18.29	0.86	12	Intermediate
Mizf	18.30	1.32	10	Intermediate
Snai3	18.30	1.11	8	Intermediate
Zfp593	18.30	2.51	18	Intermediate
Carhsp1	18.31	0.82	10	Intermediate
Mta1	18.35	0.61	12	Intermediate
Gtf2h2	18.37	1.51	6	Intermediate
Mbd3	18.38	0.76	12	Intermediate
Bloc1s1	18.39	1.38	10	Intermediate
Neurog1	18.40	1.20	21	Intermediate
Zic3	18.41	1.01	13	Intermediate
Mitf	18.41	0.95	12	Intermediate
Lin28b	18.41	3.35	18	Intermediate
Foxn4	18.41	0.89	12	Intermediate
Hey1	18.44	1.72	13	Intermediate
Zfp526	18.46	0.54	13	Intermediate
Zhx2	18.47	0.81	10	Intermediate
sox7	18.47	0.62	13	Intermediate
D330038O06Rik	18.47	0.90	10	Intermediate
Hmgb4	18.48	0.71	11	Intermediate
Zfp523	18.51	0.92	10	Intermediate
jrk	18.52	0.86	14	Intermediate
Zfp335	18.52	0.55	10	Intermediate
Stat4	18.52	1.36	10	Intermediate
Alx1	18.55	0.46	11	Intermediate
Tulp1	18.59	1.28	12	Intermediate
Dlx4	18.59	0.79	14	Intermediate
Gabpa	18.60	2.21	13	Intermediate
Zfp423	18.61	0.87	17	Intermediate
Toe1	18.63	1.07	15	Intermediate
Zfp629	18.65	1.07	10	Intermediate
Tfdp1	18.67	0.76	8	Intermediate
Tcf3	18.67	1.34	10	Intermediate
Zbtb5	18.67	0.99	10	Intermediate
Rhox9	18.68	1.36	11	Intermediate
Twist2	18.70	1.06	10	Intermediate

Tox4	18.72	0.56	17	Intermediate
E2f6	18.72	0.83	12	Intermediate
Ferd3l	18.75	1.39	10	Intermediate
Zbed4	18.77	1.14	13	Intermediate
Irx5	18.77	0.71	10	Intermediate
Thap3	18.82	1.04	11	Intermediate
Nr4a2	18.83	1.13	12	Intermediate
Prox2	18.83	1.39	24	Intermediate
Ppp1r10	18.83	1.29	11	Intermediate
Zfp42	18.85	0.83	11	Intermediate
Id3	18.85	2.99	11	Intermediate
Id2	18.86	1.24	12	Intermediate
Batf3	18.87	1.13	15	Intermediate
Brachyury	18.87	1.13	15	Intermediate
Patz1	18.87	0.38	13	Intermediate
Zc3h8	18.91	0.49	10	Intermediate
Atf7	18.92	0.70	12	Intermediate
Sp2	18.93	1.63	5	Intermediate
Tada2l	18.94	0.80	10	Intermediate
Ets2	18.95	1.09	10	Intermediate
Vsx2	18.96	0.98	10	Intermediate
Zfp9	18.97	0.89	10	Intermediate
Bhlhb4	19.01	1.17	16	Intermediate
Hoxa7	19.04	2.00	15	Intermediate
Pogz	19.06	0.89	14	Intermediate
Lhx4	19.07	1.20	13	Intermediate
Dr1	19.09	0.80	9	Intermediate
Zfp488	19.12	1.31	12	Intermediate
Foxp3	19.13	1.02	18	Intermediate
Pbx2	19.13	0.98	11	Intermediate
Tcf21	19.14	1.62	14	Intermediate
Zfp553	19.15	1.00	12	Intermediate
Lmx1b	19.15	1.75	11	Intermediate
Rreb1	19.16	0.75	15	Intermediate
Mta2	19.17	1.07	13	Intermediate
Smad3	19.26	1.55	13	Intermediate
Tcfec	19.27	1.05	11	Intermediate
Hoxa6	19.27	1.75	11	Intermediate
Hmg20a	19.31	1.31	10	Intermediate
Stat5a	19.32	2.14	10	Intermediate
Vps72	19.32	2.14	10	Intermediate
Msc	19.33	0.70	14	Intermediate
Rxrg	19.35	1.07	12	Intermediate
Trerf1	19.35	1.32	12	Intermediate
Zc3h15	19.42	2.97	10	Intermediate
Zfp568	19.43	2.15	21	Intermediate
Repin1	19.49	0.87	12	Intermediate
Edf1	19.52	1.19	16	Intermediate
Rfx3	19.60	1.44	14	Intermediate
Dmrt1	19.61	2.41	14	Intermediate
Mxd1	19.62	0.60	11	Intermediate
Neurog3	19.63	1.17	11	Intermediate
Nobox	19.64	1.31	12	Intermediate
Hopx	19.65	1.03	10	Intermediate
Klf10	19.65	1.21	11	Intermediate
Evi1	19.68	1.55	16	Intermediate
Irx4	19.70	0.88	11	Intermediate
Irf6	19.71	0.75	10	Intermediate
Nkx3-1	19.73	1.07	12	Intermediate
Zfp462	19.78	1.02	10	Intermediate
Oct4	19.80	1.56	15	Intermediate
Zfp131	19.81	0.61	13	Intermediate
Zfp280b	19.86	1.34	12	Intermediate
Fkhl18	19.87	0.34	12	Intermediate
Bhlhb2	19.87	1.13	13	Intermediate
sox8	19.96	2.46	12	Intermediate
Glis2	20.06	0.99	11	Intermediate
Hoxc5	20.07	0.76	11	Intermediate
Tcfap4	20.08	2.50	10	Intermediate
Tgif1	20.11	1.10	8	Intermediate
Lmx1a	20.13	1.80	11	Intermediate
Atf1	20.15	1.45	11	Intermediate
Tead2	20.20	1.37	12	Intermediate
Zfp641	20.21	1.01	11	Intermediate
Lhx5	20.26	1.01	12	Intermediate
Six1	20.26	1.77	11	Intermediate
Klf11	20.28	1.50	11	Intermediate
Rbpjl	20.31	0.55	20	Intermediate
Tcfe3	20.32	1.43	10	Intermediate
lef1	20.32	0.96	14	Intermediate
Maz	20.39	1.86	15	Intermediate
Ptf1a	20.45	2.86	12	Intermediate
Zfp334	20.46	0.89	10	Intermediate
Klf12	20.52	2.54	11	Intermediate
Rhox12	20.53	1.15	16	Intermediate
Zfp281	20.54	0.78	15	Intermediate
Ascl2	20.59	0.62	15	Intermediate
Ppara	20.60	3.76	14	Intermediate
Neurog2	20.69	1.66	14	Intermediate
Pdx1	20.98	0.64	13	Intermediate
Hmgb2l1	21.03	3.81	10	Intermediate
Tbx21	21.06	1.11	11	Intermediate

Pitx1	21.13	1.09	14	Intermediate
Rorc	21.17	1.56	8	Intermediate
Aatf	21.18	3.59	10	Intermediate
Otx2	21.33	1.87	6	Intermediate
Hey2	21.40	1.85	16	Intermediate
Csdc2	21.42	2.05	10	Intermediate
E2f3	21.42	2.28	16	Intermediate
Esrra	21.43	1.66	8	Intermediate
Irf3	21.43	1.87	16	Intermediate
Gatad2b	21.44	1.28	11	Intermediate
Rcor1	21.50	1.22	10	Intermediate
Tbx3	21.59	1.72	15	Intermediate
Msx1	21.66	1.53	10	Intermediate
Zic1	21.71	0.98	16	Intermediate
Pou2f2	21.76	1.27	12	Intermediate
Baz2b	21.80	0.96	12	Intermediate
Gcm2	21.86	2.22	13	Intermediate
Zfp710	21.89	1.18	10	Intermediate
Nkx2-6	21.89	1.32	10	Intermediate
Esrrb	21.91	1.12	12	Intermediate
Ets1	22.05	1.76	11	Intermediate
Hoxd3	22.10	2.12	10	Intermediate
Atf3	22.27	1.19	12	Intermediate
Pias1	22.44	1.12	13	Intermediate
Lhx6	22.56	4.95	15	Intermediate
Rhox10	22.59	0.93	10	Intermediate
Pitx2	22.68	1.63	11	Intermediate
Cdx4	22.69	0.71	19	Intermediate
Tcfap2d	22.80	1.28	12	Intermediate
Mycs	22.80	3.34	16	Intermediate
Zfp467	22.90	0.68	14	Intermediate
Myst2	22.92	1.93	11	Intermediate
Foxn3	22.94	0.87	10	Intermediate
En2	23.02	1.84	9	Enriched
Sp140	23.04	1.39	12	Enriched
Bbx	23.21	2.66	10	Enriched
Tef	23.23	1.64	7	Enriched
Hoxc10	23.27	1.26	14	Enriched
Drap1	23.46	1.06	16	Enriched
Hoxb2	23.51	1.37	11	Enriched
Nfyb	23.65	1.50	11	Enriched
Dlx3	23.66	0.89	12	Enriched
Tulp4	23.68	1.72	12	Enriched
Rxra	23.76	3.98	13	Enriched
Sp4	23.82	1.48	12	Enriched
Elf1	23.83	3.59	12	Enriched
Zfp37	24.09	1.73	11	Enriched
Nfib	24.15	3.37	12	Enriched
Ubtf	24.16	1.69	16	Enriched
Tgif2lx	24.33	0.99	13	Enriched
Gata1	24.36	2.01	13	Enriched
Zfp422	24.37	1.12	13	Enriched
Sox17	24.44	1.19	10	Enriched
Elf2	24.49	1.19	13	Enriched
Sox15	24.50	0.86	12	Enriched
Gcm1	24.52	3.30	12	Enriched
Tgif2	24.92	1.67	12	Enriched
Yy1	25.00	4.27	13	Enriched
Sfpi1	25.11	2.25	6	Enriched
Nfil3	25.20	2.32	10	Enriched
Hoxa9	25.49	4.86	24	Enriched
Nfia	25.64	2.50	10	Enriched
Zscan20	25.66	5.28	10	Enriched
Zscan4c	25.66	5.28	10	Enriched
Nfyc	25.71	2.81	12	Enriched
Pax8	25.72	1.35	12	Enriched
Barx1	26.02	1.23	7	Enriched
Sox10	26.11	3.74	10	Enriched
Barhl2	26.24	2.72	13	Enriched
Zfp524	26.31	1.95	11	Enriched
Meox2	26.85	2.56	11	Enriched
Phf20	26.91	3.91	10	Enriched
Thra	27.02	3.38	12	Enriched
Zfp41	27.20	1.96	10	Enriched
Elk3	27.27	1.53	12	Enriched
Grhl2	27.47	3.10	18	Enriched
Elk1	27.68	1.23	8	Enriched
Rhox11	28.23	1.65	14	Enriched
Irf9	28.31	1.61	10	Enriched
cbx2	28.51	4.12	13	Enriched
Six2	28.79	4.72	15	Enriched
Elf4	29.15	2.48	12	Enriched
Grhl1	29.16	6.48	10	Enriched
Ebf2	29.30	2.67	9	Enriched
Irf8	29.36	3.61	11	Enriched
Nfkb1	30.28	3.50	16	Enriched
Pax2	30.81	1.61	16	Enriched
Nr2f6	30.92	2.84	11	Enriched
Zfp280c	31.02	4.45	10	Enriched
Thap4	31.50	2.39	15	Enriched
Hoxa3	31.51	2.89	17	Enriched
Onecut2	31.67	2.75	15	Enriched

Gata2	31.78	2.32	22	Enriched
Rfx5	32.28	5.18	16	Enriched
Fli1	32.74	2.39	11	Enriched
Gata3	32.94	1.69	10	Enriched
Mafb	33.79	3.37	11	Enriched
Rfx2	34.64	2.66	13	Enriched
Creb1	34.71	6.85	8	Enriched
1700029I01Rik	35.09	6.22	11	Enriched
Smad4	35.23	10.52	15	Enriched
Ebf1	35.57	3.25	17	Enriched
Tcfap2b	36.10	2.57	13	Enriched
Tcfap2c	36.24	3.15	11	Enriched
Cdx2	37.09	7.06	13	Enriched
Irf2	37.26	3.16	10	Enriched
Vax2	37.35	4.47	12	Enriched
Hmgb2	37.49	4.21	12	Enriched
Zfp161	37.53	5.28	16	Enriched
Mbd1	38.07	5.30	11	Enriched
Hoxb13	38.55	3.34	13	Enriched
Mbd4	39.20	6.31	10	Enriched
Pou1f1	39.22	8.55	12	Enriched
hmgb3	39.94	3.37	12	Enriched
Elf5	40.02	5.03	12	Enriched
Zfp202	40.50	7.06	13	Enriched
Six6	40.68	4.30	10	Enriched
Tcfap2a	41.22	3.87	16	Enriched
Cebpe	41.58	4.60	12	Enriched
Irf4	41.80	5.24	12	Enriched
Lcor	42.21	4.70	14	Enriched
Jundm2	42.39	9.30	12	Enriched
Irf1	42.53	5.83	9	Enriched
Thap7	42.56	4.44	11	Enriched
Pias4	43.06	4.47	12	Enriched
Hmgn1	43.42	4.50	12	Enriched
Hlf	43.66	3.94	11	Enriched
Zfp319	44.44	6.21	11	Enriched
Duxbl	45.17	3.44	13	Enriched
Ebf3	45.82	6.75	10	Enriched
Dlx1	46.38	9.04	10	Enriched
Foxa1	46.84	8.55	12	Enriched
Sox2	47.70	4.86	15	Enriched
Hmgn3	50.05	4.05	19	Enriched
Nr1i2	52.11	5.19	11	Enriched
hmga2	52.62	10.20	10	Enriched
Bhlhb8	55.80	4.65	13	Enriched
Tead1	56.49	9.93	12	Enriched
Dlx6	58.39	6.25	22	Enriched
Srcap	58.60	9.45	12	Enriched
Max	65.00	9.62	18	Enriched
Mafg	69.70	11.22	17	Enriched
Zfp512	75.93	12.24	19	Enriched
Mafk	79.61	7.73	11	Enriched

Supplementary Table 3. Mitotic bound fraction measured in NIH-3T3 and E14 cells. Related to Supplementary Fig. 1.

Legend	Description
**Transcription Factor**	Name of the transcription factor
**MBF in NIH-3T3**	Mitotic bound fraction measured in NIH-3T3 cells
**SEM(MBF in NIH-3T3)**	Standard error of the mean for the mitotic bound fraction measured in NIH-3T3 cells
**n (NIH-3T3)**	Number of cells used to quantify MBF in NIH-3T3 cells
**MBF in E14 cells**	Mitotic bound fraction measured in ES cells [%]
**SEM(MBF in E14 cells)**	Standard error of the mean for the mitotic bound fraction measured in ES cells

Transcription Factor	MBF in NIH-3T3 [%]	SEM(MBF in NIH-3T3)	n (NIH-3T3)	MBF in E14 cells [%]	SEM(MBF in E14 cells)
Cdx4	20.5	0.4	12	22.7	0.7
Ebf3	27.6	3.3	6	45.8	6.8
Fam164a	14.5	0.5	10	15.1	0.6
Hmgn1	57.9	6.9	10	43.4	4.5
Mbd3	15.1	0.1	10	18.4	0.8
Nobox	23.8	1.0	10	19.6	1.3
Npas4	14.3	0.3	10	16.8	1.3
Nr0b1	14.5	0.7	10	14.1	1.8
Rbm22	16.3	0.5	10	16.6	0.7
Rhox10	18.7	0.7	11	22.6	0.9
Sox10	39.3	6.9	4	26.1	3.7
Sox15	35.2	4.2	10	24.5	0.9
Sox2	58.7	5.2	5	47.7	4.9
Thap4	43.1	3.9	10	31.5	2.4
Thap7	41.3	3.3	11	42.6	4.4
Zfp280b	20.5	0.4	10	19.9	1.3
Zfp280c	37.0	3.8	10	31.0	4.4
Zfp346	16.0	0.4	10	15.0	1.6
Zfp414	15.0	0.3	10	16.8	1.4
Zfp809	16.9	0.5	10	17.3	0.9

Supplementary Table 4. Parameters included in machine learning algorithm. Related to Fig. 1, Supplementary Fig. 1 and Methods.

**Parameters included in machine learning algorithm**	**Calculation / source**
Charge of DBD domain	Number of positively charged AA (DBD)-number of negatively charged AA(DBD)
Number of positively charged AA	protr (Xiao et al., 2015)
Number of negatively charged AA	protr (Xiao et al., 2015)
Number of neutral charged AA	protr (Xiao et al., 2015)
Length of the protein	Number of nucleotides
Charge of protein	Number of positively charged AA (protein)-number of negatively charged AA (protein)
Difference of high minus low polar AA	protr (Xiao et al., 2015)
Fraction of AA with high minus fraction of low VDW interactions	protr (Xiao et al., 2015)
Difference of AA with high minus low VDW interactions	Total VDW fraction*Length
Number of DBD	Number of domains found
Absolute charge of DBD domain per number of DBD	DBDCharge/DomainNbr
Fraction of phosphorylable AA (S, T, Y)	protr (Xiao et al., 2015)
Fraction of methylable AA (K, R)	protr (Xiao et al., 2015)
Dispersion of positive charges	Index of dispersion of positive charges in the protein (variance/mean)
Fraction of AA with a tendency to form helix structures	protr (Xiao et al., 2015)
Fraction of AA with a tendency to form strand structures	protr (Xiao et al., 2015)
Fraction of AA with a tendency to form coil structures	protr (Xiao et al., 2015)
Fraction of positive AA (K, R, H)	Lee et al., 2009
Fraction of aromatic AA (F, W, Y, H)	Lee et al., 2009
Fraction of polar AA (R, K, E, D, Q, N)	Lee et al., 2009
Fraction of hydrophobic AA (C, L, V, I, M, F, W)	Lee et al., 2009
Fraction of aliphatic AA (A, I, L, V)	Lee et al., 2009
Fraction of tiny AA (A, G)	Lee et al., 2009
Fraction of bulky AA (F, H, R, W, Y)	Lee et al., 2009
Fraction of small AA (A, G, S, T)	Lee et al., 2009
Sum of gravy coefficient	Lee et al., 2009
Number of continuous changes from positively charged AA to positively charged AA	Lee et al., 2009
Number of continuous changes from uncharged AA to uncharged AA	Lee et al., 2009
Number of continuous changes from negatively charged AA to negatively charged AA	Lee et al., 2009
Number of positively charged AA clusters	Sum of clusters of more than 2 consecutive positively charged AA

Number of uncharged AA clusters	Sum of clusters of more than 2 consecutive uncharged AA
Number of negatively charged AA clusters	Sum of clusters of more than 2 consecutive negatively charged AA
Fraction of disordered domain in the protein	Sum of ANCHOR scores / Length of the protein (Dosztnyi et al., 2009)
Number of AA in disordered domains	Sum of ANCHOR scores (Dosztnyi et al., 2009)
DBD bZIP	UNIPROT database
DBD bHLH	UNIPROT database
DBD C2H2 zinc finger	UNIPROT database
DBD HMG	UNIPROT database
DBD Nuclear receptors with C4 zinc fingers	UNIPROT database
DBD T Box	UNIPROT database
DBD C2HC zinc finger	UNIPROT database
DBD SMAD NF1 DBD	UNIPROT database
DBD DM type intertwined zinc finger	UNIPROT database
DBD Homeo domain	UNIPROT database
DBD Tryptophan cluster	UNIPROT database
DBD C3H zinc finger	UNIPROT database
DBD TEA domain	UNIPROT database
DBD Fork head winged helix	UNIPROT database
DBD RHR	UNIPROT database
DBD Heteromeric CCAAT binding	UNIPROT database
DBD Other C4 zinc finger type	UNIPROT database
DBD GCM domain	UNIPROT database
DBD ARID domain	UNIPROT database
DBD Grainyhead domain	UNIPROT database
DBD MADS box	UNIPROT database
DBD Cold shock domain	UNIPROT database
DBD CXXC zinc finger	UNIPROT database
DBD C2CH THAP type zinc finger	UNIPROT database

Supplementary Table 5. Correlation between Hoechst and YPet signals in the whole cell, in heterochromatin, DNA-rich, and DNA-poor regions. Related to Fig. 2.

Legend	Description
**Transcription Factor**	Name of the transcription factor
**Pixel_Corr_HoechstYpet**	Pixel by pixel correlation between Hoechst and YPet signals (whole cell)
**SEM_Pixel_Corr_HoechstYpet**	Standard error of the mean for the pixel by pixel correlation between Hoechst and YPet signals
**Heterochromatin**	Correlation between Hoechst and YPet signals in heterochromatin
**SEM_Heterochromatin**	Standard error of the mean for the correlation between Hoechst and YPet signals in heterochromatin
**DNA-rich**	Correlation between Hoechst and YPet signals in DNA-rich regions
**SEM_DNA-rich**	Standard error of the mean for the correlation between Hoechst and YPet signals in DNA-rich regions
**DNA-poor**	Correlation between Hoechst and YPet signals in DNA-poor regions
**SEM_DNA-poor**	Standard error of the mean for the correlation between Hoechst and YPet signals in DNA-poor regions

Transcription Factor	Pixel_Corr_HoechstYpet	SEM_Pixel_Corr_HoechstYpet	Heterochromatin	SEM_Heterochromatin	DNA-rich	SEM_DNA-rich	DNA-poor	SEM_DNA-poor
Atf3	0.56	0.06	0.17	0.02	0.27	0.01	0.56	0.04
Bhlhb8	0.76	0.02	0.19	0.01	0.34	0.01	0.47	0.01
Brachyury	0.36	0.05	0.08	0.01	0.34	0.01	0.58	0.02
CDX2	0.39	0.06	0.05	0.00	0.39	0.01	0.57	0.02
Dlx1	0.53	0.04	0.12	0.00	0.37	0.01	0.51	0.03
Dlx6	0.70	0.02	0.20	0.01	0.34	0.00	0.46	0.01
Duxbl	0.84	0.02	0.28	0.01	0.30	0.01	0.42	0.02
Ebf1	0.35	0.04	0.05	0.00	0.38	0.01	0.58	0.02
Foxa1	0.16	0.08	0.05	0.00	0.39	0.02	0.56	0.02
Gata5	0.30	0.06	0.07	0.00	0.31	0.01	0.62	0.04
Hes3	0.36	0.06	0.07	0.01	0.27	0.01	0.66	0.03
Hlf	0.13	0.07	0.05	0.01	0.31	0.01	0.65	0.02
Hmga2	0.77	0.02	0.19	0.00	0.27	0.00	0.54	0.03
Hmgb2	0.80	0.02	0.17	0.01	0.29	0.00	0.54	0.02
Hmgn1	0.71	0.02	0.14	0.01	0.36	0.00	0.50	0.01
Hoxd10	0.39	0.05	0.11	0.02	0.31	0.01	0.58	0.03
Mafg	0.57	0.05	0.21	0.01	0.33	0.00	0.46	0.02
Mafk	0.62	0.06	0.24	0.01	0.33	0.01	0.43	0.02
Max	0.61	0.05	0.08	0.01	0.40	0.00	0.52	0.02
Nanog	0.33	0.05	0.09	0.01	0.32	0.01	0.59	0.02
Nr1i2	0.18	0.04	0.07	0.01	0.30	0.01	0.63	0.01
Pax2	0.04	0.03	0.07	0.01	0.32	0.01	0.61	0.02
Pias4	0.53	0.04	0.12	0.01	0.38	0.01	0.51	0.02
Pou1f1	0.33	0.07	0.12	0.01	0.36	0.00	0.52	0.02
Oct4	0.61	0.03	0.15	0.01	0.35	0.00	0.50	0.03
Prr3	0.32	0.07	0.05	0.01	0.31	0.01	0.63	0.04
Rhox11	0.37	0.08	0.08	0.00	0.31	0.01	0.61	0.03
Six6	0.39	0.06	0.12	0.01	0.34	0.01	0.54	0.01
Sox15	0.49	0.04	0.10	0.01	0.39	0.01	0.51	0.02
Sox2	0.35	0.05	0.07	0.00	0.38	0.00	0.55	0.02
Srcap	0.71	0.03	0.16	0.01	0.33	0.01	0.52	0.01
Tcfec	0.41	0.06	0.14	0.06	0.30	0.01	0.56	0.06
Tead1	0.86	0.01	0.26	0.02	0.33	0.00	0.41	0.02
Thap4	0.37	0.05	0.05	0.00	0.37	0.02	0.58	0.02
Tox3	0.36	0.06	0.04	0.00	0.31	0.01	0.65	0.04
Zfp131	0.26	0.07	0.05	0.00	0.27	0.01	0.68	0.05
Zfp319	0.34	0.05	0.09	0.01	0.36	0.02	0.56	0.02
Zmat2	0.43	0.07	0.08	0.00	0.23	0.01	0.68	0.03

Supplementary Table 6. Half-times of FRAP recovery in mitosis and interphase cells and predicted molecular weight. Related to Fig. 3 and Supplementary Fig. 2.

Legend	Description
**Transcription Factor**	Name of the transcription factor
**FRAP t1/2 recovery time inter.**	Interphase half time of fluorescence recovery
**SEM FRAP t1/2 recovery time inter.**	Standard error of the interphase half time of fluorescence recovery
**FRAP t1/2 recovery time meta.**	Mitotic half time of fluorescence recovery
**SEM FRAP t1/2 recovery time meta.**	Standard error of the mitotic half time of fluorescence recovery
**FRAP immobile fraction inter.**	Interphase FRAP immobile fraction
**SEM FRAP immobile fraction inter.**	Standard error of the interphase FRAP immobile fraction
**FRAP immobile fraction mitosis**	Mitotic FRAP immobile fraction
**SEM FRAP immobile fraction mitosis**	Standard error of the mitotic FRAP immobile fraction
**Predicted Molecular Weight [kDa]**	Predicted Molecular Weight, based on protein and YPet tag amino acid sequence

**Transcription Factor**	**FRAP t1/2 recovery time inter. [s]**	**SEM FRAP t1/2 recovery time inter.**	**FRAP t1/2 recovery time meta. [s]**	**SEM FRAP t1/2 recovery time meta.**	**FRAP immobile fraction inter.**	**SEM FRAP immobile fraction inter.**	**FRAP immobile fraction mitosis**	**SEM FRAP immobile fraction mitosis**	**Predicted Molecular Weight [kDa]**
BHLHB8	10.82	1.03	13.48	1.66	0.08	0.03	0.09	0.02	51.78
BRACHYURY	4.20	0.45	NA	NA	0.09	0.03	NA	NA	77.08
CDX2	14.03	1.09	7.78	0.93	0.21	0.02	0.18	0.03	63.11
DLX6	6.91	0.75	13.25	1.71	0.10	0.03	0.05	0.01	49.22
FOXA1	6.47	0.59	3.87	0.41	0.13	0.02	0.11	0.04	78.49
HMGA2	9.18	1.23	10.59	5.57	0.11	0.04	0.12	0.05	41.45
HMGB2	7.87	1.61	1.21	0.11	0.08	0.03	0.08	0.03	53.80
HMGN1	4.57	0.58	5.40	0.64	0.10	0.01	0.04	0.02	39.78
HOXD10	3.50	0.92	1.71	0.52	0.03	0.01	0.05	0.03	67.96
MAFK	17.95	3.10	19.33	4.34	0.20	0.05	0.19	0.07	47.17
MAX	16.21	1.59	30.86	5.39	0.22	0.04	0.23	0.07	46.80
NANOG	3.55	0.40	NA	NA	0.03	0.01	NA	NA	63.88
POU5F1	5.33	0.47	4.70	1.93	0.08	0.02	0.08	0.02	67.85
SOX15	2.92	0.37	2.03	0.22	0.04	0.01	0.02	0.01	54.95
SOX2	7.68	0.80	9.97	0.60	0.12	0.03	0.19	0.04	64.04
TEAD1	8.51	1.11	4.60	0.80	0.10	0.04	0.07	0.03	76.43
THAP4	2.29	0.53	2.80	0.47	0.10	0.06	0.09	0.03	92.21
ZFP131	4.45	0.58	NA	NA	0.10	0.03	NA	NA	96.54

Supplementary Table 7. Pseudo ON-rates and relative residence times per transcription factor. Related to Fig. 3, Fig. 4 and Supplementary Fig. 3.

**Legend**	**Description**
**Transcription Factor**	Name of the transcription factor
**Heterochromatin ΨON-rate**	Pseudo on-rate in heterochromatin
**DNA-rich ΨON-rate**	Pseudo on-rate in DNA-rich regions
**DNA-poor ΨON-rate**	Pseudo on-rate in DNA-poor regions
**Sum ΨON-rate (total)**	Sum of pseudo on-rate over the whole nucleus
**Average residence times (Total)**	Relative residence time of each transcription factor

**Transcription Factor**	**Heterochromatin ΨON-rate [A.U.]**	**DNA-rich ΨON-rate [A.U.]**	**DNA-poor ΨON-rate [A.U.]**	**Sum ΨON-rate (total) [A.U.]**	**Average residence times (Total) [A.U.]**
Foxa1	1.32E-05	1.43E-05	1.46E-05	4.21E-05	6.5047
Nanog	1.11E-05	1.57E-05	1.96E-05	4.64E-05	8.83455
Sox15	1.57E-05	2.01E-05	1.70E-05	5.27E-05	7.70267
Hoxd10	1.82E-05	1.98E-05	1.98E-05	5.79E-05	7.84462
Tead1	1.87E-05	2.29E-05	1.68E-05	5.85E-05	7.84896
Cdx2	1.42E-05	2.66E-05	2.04E-05	6.12E-05	9.16559
Dlx6	2.59E-05	2.18E-05	1.96E-05	6.73E-05	7.98
Sox2	2.58E-05	2.97E-05	2.17E-05	7.71E-05	7.81308
Bhlhb8	4.59E-05	4.16E-05	2.22E-05	0.00011	7.015

Supplementary Table 8. Number of peaks called, fraction of reads in peaks and fraction of occupied motifs in close and open genome per TF. Related to Fig. 4, Fig. 5, Supplementary Fig. 3, Supplementary Fig. 4 and Supplementary Fig. 5.

**Legend**	**Description**
**Transcription Factor**	Name of the transcription factor
**HA_intensity**	Expression levels assessed by quantitative analysis of immunofluorescence images normalized to background
**MACS2**	Number of peaks called with MACS2
**MACS2_q0.01**	Number of peaks called with MACS2 (q<0.01)
**HOMER**	Number of peaks called with HOMER
**MACS2_downsampled**	Number of peaks called with MACS2 (downsampling to 10^7 total reads for each TF)
**FRiP [%]**	Fraction of reads in peaks
**FOM_all**	Fraction of occupied top motifs
**FOM_open**	Fraction of occupied top motifs in open genome
**FOM_closed**	Fraction of occupied top motifs in closed genome
**FOM_published**	Fraction of occupied published motifs
**FOM_published_open**	Fraction of occupied published motifs in open genome
**Fraction_peaks_in_closed [%]**	Fraction of ChIP peaks in closed chromatin

**Transcription Factor**	**HA_intensity [A.U.]**	**MACS2**	**MACS2_q0.01**	**HOMER**	**MACS2_downsampled**	**FRiP [%]**	**FOM_all [%]**	**FOM_open [%]**	**FOM_closed [%]**	**FOM_published [%]**	**FOM_published_open [%]**	**Fraction_peaks_in_closed [%]**
BHLHB8_1	7.96	46842	39556	8403	5792	12.40	1.97	33.59	1.29	NA	NA	41.32
BHLHB8_2	7.96	61991	39588	15722	10359	21.49	3.50	22.73	2.17	NA	NA	62.48
BRACHYURY	3.27	720	456	25	2	0.10	0.05	0.68	0.26	0.02	2.43	22.78
CDX2	10.85	25278	18012	2793	1079	4.21	1.81	6.97	1.98	1.12	30.62	83.84
DLX1	6.56	20124	14475	1543	452	3.42	1.36	23.22	0.18	0.19	35.97	19.78
DLX6	3.05	20983	16007	4033	2034	4.98	1.55	25.34	0.14	0.23	35.11	21.11
DUXBL	4.80	24318	17292	8328	4430	5.54	7.90	8.21	7.30	7.90	65.85	78.16
EBF1	3.57	7669	6421	2894	1205	2.37	1.08	32.15	0.48	1.08	41.75	20.43
FOXA1_1	3.19	6033	4750	1406	417	1.23	0.18	20.56	0.10	NA	NA	26.14
FOXA1_2	3.19	1099	783	237	30	0.21	0.06	6.28	0.01	NA	NA	26.48
FOXA1_RR	6.31	151	101	17	6	0.03	0.01	1.58	0.02	0.00	0.38	25.17
FOXA1_SW	5.82	147	93	4	3	0.03	0.02	6.56	0.03	NA	NA	38.78
HLF	1.91	2635	1934	288	45	0.38	0.12	19.05	0.09	0.06	5.97	26.76
MAX	5.29	3836	3070	2075	655	1.21	0.31	16.84	0.15	NA	NA	27.22
NANOG	3.16	396	269	6	1	0.05	0.02	0.79	0.02	NA	NA	32.58
POU5F1_1	2.82	95	68	24	4	0.02	0.01	1.23	0.01	NA	NA	17.89
POU5F1_2	2.82	52	33	0	1	0.01	0.01	0.58	0.34	NA	NA	15.38
PRR3	3.46	62	41	1	2	0.02	0.11	7.69	0.18	NA	NA	32.26
RHOX11	1.79	4579	2833	162	28	0.62	2.66	11.18	0.08	0.05	3.49	35.60
SIX6	3.07	6075	4727	970	343	1.16	0.37	6.53	1.54	0.07	11.61	21.23
SOX15	5.03	5002	3935	1360	637	1.26	0.34	6.28	0.02	0.10	9.81	14.25
SOX2_1	3.86	25049	21918	7236	5223	7.99	1.52	30.01	0.15	NA	NA	26.14
SOX2_2	3.86	5692	4628	1844	849	1.47	0.40	7.04	0.05	NA	NA	18.10
TEAD1	3.79	14959	10606	27424	10903	9.53	NA	69.06	11.02	NA	NA	65.49
THAP4	3.31	58	40	9	5	0.02	1.74	17.65	0.11	NA	NA	27.59
TOX3	2.28	1286	1026	416	166	0.43	0.04	0.69	0.14	NA	NA	8.40
ZFP319	2.06	14645	11914	7825	4958	7.96	NA	42.20	5.72	NA	NA	78.34

Supplementary Table 9. Motifs enriched in ChIP-seq peaks for each TF. Related to Fig. 4, Supplementary Fig. 4, Supplementary Fig. 5 and methods.

**Legend**	**Description**
**Transcription Factor**	Name of the transcription factor
**Motif_all_peaks**	Most highly enriched de novo motif in ChIP-seq peaks
**Motif_open_peaks**	Most highly enriched de novo motif in ChIP-seq peaks in accessible regions
**Motif_closed_peaks**	Most highly enriched de novo motif in ChIP-seq peaks in non-accessible regions
**Published_motif**	Enriched published motif for the corresponding transcription factor or a closely related protein

**Transcription Factor**	**Motif_all_peaks**	**Motif_open_peaks**	**Motif_closed_peaks**	**Published_motif**
BHLHB8_1	Atoh1(bHLH)/Cerebellum-Atoh1-ChIP-Seq(GSE22111)/Homer(0.946)	Atf3(bZIP)/GBM-ATF3-ChIP-Seq(GSE33912)/Homer(0.992)	MA0091.1_TAL1::TCF3/Jaspar(0.947)	Olig2(bHLH)/Neuron-Olig2-ChIP-Seq(GSE30882)/Homer
BHLHB8_2	MA0091.1_TAL1::TCF3/Jaspar(0.940)	AP-1(bZIP)/ThioMac-PU.1-ChIP-Seq(GSE21512)/Homer(0.995)	Atoh1(bHLH)/Cerebellum-Atoh1-ChIP-Seq(GSE22111)/Homer(0.967)	Olig2(bHLH)/Neuron-Olig2-ChIP-Seq(GSE30882)/Homer
BRACHYURY	AP-1(bZIP)/ThioMac-PU.1-ChIP-Seq(GSE21512)/Homer(0.987)	Fra1(bZIP)/BT549-Fra1-ChIP-Seq(GSE46166)/Homer(0.984)	PB0117.1_Eomes_2/Jaspar(0.742)	Brachyury(T-box)/Mesoendoderm-Brachyury-ChIP-exo(GSE54963)/Homer
CDX2	caudal(Homeobox)/Drosophila-Embryos-ChIP-Chip(modEncode)/Homer(0.959)	Atf3(bZIP)/GBM-ATF3-ChIP-Seq(GSE33912)/Homer(0.995)	MA0465.1_CDX2/Jaspar(0.958)	Cdx2(Homeobox)/mES-Cdx2-ChIP-Seq(GSE14586)/Homer
DLX1	Atf3(bZIP)/GBM-ATF3-ChIP-Seq(GSE33912)/Homer(0.991)	Fra1(bZIP)/BT549-Fra1-ChIP-Seq(GSE46166)/Homer(0.994)	MA0091.1_TAL1::TCF3/Jaspar(0.934)	PH0020.1
DLX6	AP-1(bZIP)/ThioMac-PU.1-ChIP-Seq(GSE21512)/Homer(0.992)	Fra1(bZIP)/BT549-Fra1-ChIP-Seq(GSE46166)/Homer(0.993)	RUNX(Runt)/HPC7-Runx1-ChIP-Seq(GSE22178)/Homer(0.971)	MA0882.1
DUXBL	MA0468.1_DUX4/Jaspar(0.962)	Fra1(bZIP)/BT549-Fra1-ChIP-Seq(GSE46166)/Homer(0.981)	MA0468.1_DUX4/Jaspar(0.903)	MA0468.1_DUX4/Jaspar(0.962)
EBF1	EBF(EBF)/proBcell-EBF-ChIP-Seq(GSE21978)/Homer(0.994)	EBF(EBF)/proBcell-EBF-ChIP-Seq(GSE21978)/Homer(0.969)	EBF(EBF)/proBcell-EBF-ChIP-Seq(GSE21978)/Homer(0.989)	EBF(EBF)/proBcell-EBF-ChIP-Seq(GSE21978)/Homer(0.994)
FOXA1_1	Foxa2(Forkhead)/Liver-Foxa2-ChIP-Seq(GSE25694)/Homer(0.972)	MA0047.2_Foxa2/Jaspar(0.974)	MA0047.2_Foxa2/Jaspar(0.974)	Foxa2(Forkhead)/Liver-Foxa2-ChIP-Seq(GSE25694)/Homer
FOXA1_2	MA0047.2_Foxa2/Jaspar(0.983)	Foxa2(Forkhead)/Liver-Foxa2-ChIP-Seq(GSE25694)/Homer(0.975)	Foxa2(Forkhead)/Liver-Foxa2-ChIP-Seq(GSE25694)/Homer(0.977)	Foxa2(Forkhead)/Liver-Foxa2-ChIP-Seq(GSE25694)/Homer
FOXA1_RR	MA0031.1_FOXD1/Jaspar(0.885)	MA0047.2_Foxa2/Jaspar(0.832)	vnd/dmmpmm(Noyes_hd)/fly(0.666)	Foxa2(Forkhead)/Liver-Foxa2-ChIP-Seq(GSE25694)/Homer
FOXA1_SW	SIP4/SIP4_SM/9-SIP4(Harbison)/Yeast(0.761)	Deaf1/dmmpmm(Bigfoot)/fly(0.668)	GAGA-repeat/Arabidopsis-Promoters/Homer(0.712)	NA
HLF	MF0006.1_bZIP_cEBP-like_subclass/Jaspar(0.903)	MF0006.1_bZIP_cEBP-like_subclass/Jaspar(0.922)	MA0025.1_NFIL3/Jaspar(0.905)	MA0043.1
MAX	MA0047.2_Foxa2/Jaspar(0.982)	MA0047.2_Foxa2/Jaspar(0.986)	MA0047.2_Foxa2/Jaspar(0.941)	NA
NANOG	RUNX-AML(Runt)/CD4+-PolII-ChIP-Seq(Barski et al.)/Homer(0.964)	RUNX2(Runt)/PCa-RUNX2-ChIP-Seq(GSE33889)/Homer(0.866)	PRDM1(Zf)/Hela-PRDM1-ChIP-Seq(GSE31477)/Homer(0.669)	NA
OCT4_1	Pit1(Homeobox)/GCrat-Pit1-ChIP-Seq(GSE58009)/Homer(0.903)	Pit1(Homeobox)/GCrat-Pit1-ChIP-Seq(GSE58009)/Homer(0.873)	Eip74EF/dmmpmm(SeSiMCMC)/fly(0.615)	Oct4(POU,Homeobox)/mES-Oct4-ChIP-Seq(GSE11431)/Homer
OCT4_2	Srebp2(bHLH)/HepG2-Srebp2-ChIP-Seq(GSE31477)/Homer(0.790)	MATA1(MacIsaac)/Yeast(0.629)	YDR026c/YDR026c_YPD/[](Harbison)/Yeast(0.778)	Oct4(POU,Homeobox)/mES-Oct4-ChIP-Seq(GSE11431)/Homer
PRR3	Srebp1a(bHLH)/HepG2-Srebp1a-ChIP-Seq(GSE31477)/Homer(0.638)	HAP2/Literature(Harbison)/Yeast(0.640)	STAT6(Stat)/Macrophage-Stat6-ChIP-Seq(GSE38377)/Homer(0.750)	NA
RHOX11	GFY(?)/Promoter/Homer(0.953)	Elk4(ETS)/Hela-Elk4-ChIP-Seq(GSE31477)/Homer(0.985)	ovo/dmmpmm(Down)/fly(0.702)	PH0158.1_Rhox11_2/Jaspar(0.834)
SIX6	Fosl2(bZIP)/3T3L1-Fosl2-ChIP-Seq(GSE56872)/Homer(0.990)	Fra1(bZIP)/BT549-Fra1-ChIP-Seq(GSE46166)/Homer(0.993)	MA0426.1_YHP1/Jaspar(0.715)	PB0059.1
SOX15	Fra1(bZIP)/BT549-Fra1-ChIP-Seq(GSE46166)/Homer(0.990)	Fra1(bZIP)/BT549-Fra1-ChIP-Seq(GSE46166)/Homer(0.988)	Sox2(HMG)/mES-Sox2-ChIP-Seq(GSE11431)/Homer(0.956)	Sox3(HMG)/NPC-Sox3-ChIP-Seq(GSE33059)/Homer
SOX2_1	Atf3(bZIP)/GBM-ATF3-ChIP-Seq(GSE33912)/Homer(0.992)	Atf3(bZIP)/GBM-ATF3-ChIP-Seq(GSE33912)/Homer(0.991)	Sox3(HMG)/NPC-Sox3-ChIP-Seq(GSE33059)/Homer(0.976)	Sox3(HMG)/NPC-Sox3-ChIP-Seq(GSE33059)/Homer
SOX2_2	Fra1(bZIP)/BT549-Fra1-ChIP-Seq(GSE46166)/Homer(0.993)	Fra1(bZIP)/BT549-Fra1-ChIP-Seq(GSE46166)/Homer(0.993)	Sox3(HMG)/NPC-Sox3-ChIP-Seq(GSE33059)/Homer(0.966)	Sox3(HMG)/NPC-Sox3-ChIP-Seq(GSE33059)/Homer
TEAD1	SeqBias: CG-repeat(0.787)	PHD1/PHD1_BUT90/8-SUT1(Harbison)/Yeast(0.781)	PB0095.1_Zfp161_1/Jaspar(0.804)	NA
THAP4	SPL14(SBP)/Arabidopsis thaliana/AthaMap(0.776)	PB0086.1_Tcfap2b_1/Jaspar(0.747)	twi/dmmpmm(Papatsenko)/fly(0.741)	NA
TOX3	AP-1(bZIP)/ThioMac-PU.1-ChIP-Seq(GSE21512)/Homer(0.978)	AP-1(bZIP)/ThioMac-PU.1-ChIP-Seq(GSE21512)/Homer(0.969)	br-Z1/dmmpmm(Bigfoot)/fly(0.662)	NA
ZFP319	SeqBias: GA-repeat(0.900)	Trl/dmmpmm(Down)/fly(0.905)	Trl/dmmpmm(Down)/fly(0.852)	NA

Supplementary Table 10. Number and fraction of TF ChIP-seq peaks and ATAC-seq peaks with changed chromatin accessibility per TF. Related to Fig. 5 and Supplementary Fig. 5.

**Legend**	**Description**
**Transcription Factor**	Name of the transcription factor
**nrpeaks_merged**	Number of merged ChIP-seq peaks
**TFsites_changed**	Number of ChIP-seq peaks with changed chromatin accessibility
**TFsites_up**	Number of ChIP-seq peaks with increased chromatin accessibility
**TFsites_down**	Number of ChIP-seq peaks with decreased chromatin accessibility
**TFsites_fraction_changed**	Fraction of TF peaks with changed accessibility
**TFsites_fraction_up**	Fraction of TF peaks with increased accessibility
**TFsites_fraction_down**	Fraction of TF peaks with decreased accessibility
**ATACsites_changed**	Number of ATAC-seq peaks with changed chromatin accessibility
**ATACsites_up**	Number of ATAC-seq peaks with increased chromatin accessibility
**ATACsites_down**	Number of ATAC-seq peaks with decreased chromatin accessibility

**Transcription Factor**	**nrpeaks_merged**	**TFsites_changed**	**TFsites_up**	**TFsites_down**	**TFsites_fraction_changed [%]**	**TFsites_fraction_up [%]**	**TFsites_fraction_down [%]**	**ATACsites_changed**	**ATACsites_up**	**ATACsites_down**
BHLHB8	82754	11	7	4	0.01	0.01	0.00	7	6	1
BRACHYURY	708	1	1	0	0.14	0.14	0.00	12	11	1
CDX2	25190	11652	11418	234	46.26	45.33	0.93	26937	16162	10775
DLX6	20563	11	11	0	0.05	0.05	0.00	22	18	4
DUXBL	24265	303	7	296	1.25	0.03	1.22	1280	315	965
FOXA1	6201	783	726	57	12.63	11.71	0.92	7913	5539	2374
HLF	2601	0	0	0	0.00	0.00	0.00	2	2	0
NANOG	383	1	1	0	0.26	0.26	0.00	5	4	1
POU5F1	116	0	0	0	0.00	0.00	0.00	8	8	0
RHOX11	4533	14	4	10	0.31	0.09	0.22	3374	2322	1052
SIX6	5976	94	1	93	1.57	0.02	1.56	249	73	176
SOX15	4934	2	2	0	0.04	0.04	0.00	57	51	6
SOX2	25045	1849	1704	145	7.38	6.80	0.58	7438	5549	1889

Supplementary Table 11. Primers used for PCR cloning

Construct	Primer name	Sequence
Gbx2 pENTR	Gbx2-F	5'-ggggacaagtttgtacaaaaaagcaggcaccatgagcgcagcgttcc-3'
	Gbx2-R	5'-ggggaccactttgtacaagaaagctgggtcgggtcgggcctgctc-3'
Klf2 pENTR	Klf2-F	5'-ggggacaagtttgtacaaaaaagcaggcaccatggcgctcagcgagc-3'
	Klf2-R	5'-ggggaccactttgtacaagaaagctgggtccatgtgtcgcttcatgtgc-3'
Klf4 pENTR	Klf4-F	5'-ggggacaagtttgtacaaaaaagcaggcaccatgaggcagccacctgg-3'
	Klf4-R	5'-ggggaccactttgtacaagaaagctgggtcaaagtgcctcttcatgtgtaagg-3'
Klf5 pENTR	Klf5-F	5'-ggggacaagtttgtacaaaaaagcaggcaccatgcccacgcgggtg-3'
	Klf5-R	5'-ggggaccactttgtacaagaaagctgggtcgttctggtggcgcttcat-3'
Nanog pENTR	Nanog-F	5'-ggggacaagtttgtacaaaaaagcaggcaccatgagtgtgggtcttcctgg-3'
	Nanog-R	5'-ggggaccactttgtacaagaaagctgggtctatttcacctggtggagtcaca-3'
sox17 pENTR	sox17-F	5'-ggggacaagtttgtacaaaaaagcaggcaccatgagcagcccggatg-3'
	sox17-R	5'-ggggaccactttgtacaagaaagctgggtcaatgtcggggtagttgcaat-3'
Tcf3 pENTR	Tcf3-F	5'-ggggacaagtttgtacaaaaaagcaggcaccatgccccagctcggtg-3'
	Tcf3-R	5'-ggggaccactttgtacaagaaagctgggtcgtgggcagacttggtgacc-3'
FoxA1_SW	FoxA1SWmut-F	5’-ccaggcaagggcgcctacgcgacgctgcaccc-3’
FoxA1_RR	FoxA1RRmut-F	5’-cggctgctacttgcgcgcccaaaaggccttcaagtgtgagaag-3’
YPet-NLS (all)	YPet-NLS-F	5’-tttgtcgacatggtgagcaaaggcgaa-3’
NLS(RKRKR)	YPet-RKRKR-R	5’-tttcatatgttatctcttccgttttcgcttatagagctcgttcatgcc-3’
YPet-RRRRR	YPet-RRRRR-R	5’-tttcatatgttaccttctcctgcgcctcttatagagctcgttcatgcc-3’
YPet-RKARKR	YPet-RKARKR-R	5’-tttcatatgttatctcttccgagcttttcgcttatagagctcgttcatgcc-3’
YPet-RKARKAR	YPet-RKARKAR-R	5’-tttcatatgttatctagccttccgagcttttcgcttatagagctcgttcatgcc-3’
YPet	YPet-R	5’-tttcatatgttacttatagagctcgttcatgcc-3’
5R-OCT4	5R-OCT4-F	5’-ggggacaagtttgtacaaaaaagcaggctctatgagaaggaggaggagaatggctggacacctggc-3’
	5R-OCT4-R	5’-ggggctttgtacaagaaagctgggtcgtttgaatgcatgggagagcc-3’
5R-BRACHYURY	5R-BRA-F	5’-ggggacaagtttgtacaaaaaagcaggctctatgagaaggaggaggagaatgagctcgccgggcacagag-3’
	5R-BRA-R	5’-ggggaccactttgtacaagaaagctgggtccatggatgggggtgacaca-3’
5R-NANOG	5R-NANOG-F	5’- ggggacaagtttgtacaaaaaagcaggctctatgagaaggaggaggagaatgagtgtgggtcttcctgg-3’
	5R-NANOG-R	5’-ggggaccactttgtacaagaaagctgggtctatttcacctggtggagtcaca-3’
5R-PRR3	5R-PRR3-F	5’-ggggacaagtttgtacaaaaaagcaggctctatgagaaggaggaggagaatggctaacgggaagcctg-3’
	5R-PRR3-R:	5’-ggggaccactttgtacaagaaagctgggtccagaggaggtccgttgacg-3’
