# Supplementary material for: Mitotic chromosome binding predicts transcription factor properties in interphase
Source: Nat Commun. 2019 Jan 30;10:487. doi: 10.1038/s41467-019-08417-5 (PMC6353955; doi:10.1038/s41467-019-08417-5)
Supplement: Supplementary file 3 — Reporting Summary [file 41467_2019_8417_MOESM3_ESM.pdf]

nature research

Corresponding author(s): David Suter

Reporting Summary

Nature Research wishes to improve the reproducibility of the work that we publish. This form provides structure for consistency and transparency in reporting. For further information on Nature Research policies, see Authors & Referees and the Editorial Policy Checklist.

Statistical parameters

When statistical analyses are reported, confirm that the following items are present in the relevant location (e.g. figure legend, table legend, main text, or Methods section).

n/a	Confirmed	
☐	☒	The exact sample size (n) for each experimental group/condition, given as a discrete number and unit of measurement
☐	☒	An indication of whether measurements were taken from distinct samples or whether the same sample was measured repeatedly
☐	☒	The statistical test(s) used AND whether they are one- or two-sided<br>*Only common tests should be described solely by name; describe more complex techniques in the Methods section.*
☐	☒	A description of all covariates tested
☒	☐	A description of any assumptions or corrections, such as tests of normality and adjustment for multiple comparisons
☐	☒	A full description of the statistics including central tendency (e.g. means) or other basic estimates (e.g. regression coefficient) AND variation (e.g. standard deviation) or associated estimates of uncertainty (e.g. confidence intervals)
☐	☒	For null hypothesis testing, the test statistic (e.g. F, t, r) with confidence intervals, effect sizes, degrees of freedom and P value noted<br>*Give P values as exact values whenever suitable.*
☒	☐	For Bayesian analysis, information on the choice of priors and Markov chain Monte Carlo settings
☒	☐	For hierarchical and complex designs, identification of the appropriate level for tests and full reporting of outcomes
☐	☒	Estimates of effect sizes (e.g. Cohen's d, Pearson's r), indicating how they were calculated
☐	☒	Clearly defined error bars<br>*State explicitly what error bars represent (e.g. SD, SE, CI)*

Our web collection on statistics for biologists may be useful.

Software and code

Policy information about availability of computer code

Data collection	The mitotic bound fractions were imaged using IN Cell Analyzer 2200 V7.1.<br>FRAP and Hoechst-YPet colocalization were imaged using ZEN 2009 software.<br>Single molecule image collection was performed as described in Clauß et al., 2017
Data analysis	Statistics were calculated using R Studio 1.0.153. R packages used are: GenomicRanges_1.28.6, edgeR_3.18.1, lima_3.32.10, ggplot2_3.0.0. The following software was used for ATAC-seq and ChIP-seq analysis: STAR 2.5.3a, SAMTools 1.4, picard 2.8.3, HOMER 4.7, deepTools 2.4.2, BEDTools 2.2.6.0., MACS 2.1.1.20160309.<br>For protein sequence analysis and machine learning analysis, R studio 1.0.143 was used with the following R packages: glmnet 2.0-16, gridExtra 2.3, stringr 1.3.1, data.table 1.11.4, ggplot2 3.0.0, Hmisc 4.1-1, protr 1.5-1, scales 0.5.0. Disordered domains were identified using online ANCHOR software on 26.09.2018.<br>FRAP data were analyzed using the Matlab standalone version of easyFRAP R2015b (9.0). Hoechst-YPet colocalization were analyzed using FIJI from ImageJ 2.0.0-rc-43. The mitotic bound fractions were quantified using a semi-automated pipeline on CellProfiler 2.1.1.<br>Single molecule image analysis was performed using a self-written code in Matlab R2017b.

For manuscripts utilizing custom algorithms or software that are central to the research but not yet described in published literature, software must be made available to editors/reviewers upon request. We strongly encourage code deposition in a community repository (e.g. GitHub). See the Nature Research guidelines for submitting code & software for further information.

Data

Policy information about availability of data

All manuscripts must include a data availability statement. This statement should provide the following information, where applicable:
- Accession codes, unique identifiers, or web links for publicly available datasets
- A list of figures that have associated raw data
- A description of any restrictions on data availability

ChIP-seq and ATAC-seq data that support the findings of this study have been deposited in GEO (Gene Expression Omnibus) with the accession code GSE119784 (https://www.ncbi.nlm.nih.gov/geo/query/acc.cgi?acc=GSE119784, reviewer access token: “uxklyewcdbyjdir”). This raw data is used in Figures 4a-e, Figures 5a-e, Supplementary Figure 3d-f, Supplementary Figures 4a-d/f-j, Supplementary Figures 5a-g.

Field-specific reporting

Please select the best fit for your research. If you are not sure, read the appropriate sections before making your selection.

☒ Life sciences ☐ Behavioural & social sciences ☐ Ecological, evolutionary & environmental sciences

For a reference copy of the document with all sections, see nature.com/authors/policies/ReportingSummary-flat.pdf

Life sciences study design

All studies must disclose on these points even when the disclosure is negative.

Sample size	No sample size calculation was performed. Due to fairly constant values of Mitotic Bound Fraction between cells per clone, we believe that the number of cells quantified and the achieved statistical significance support our conclusions.
Data exclusions	The DNA binding domain families were excluded from the machine learning algorithm if present in less than 10 TFs for which we obtained a value of Mitotic Bound Fraction.
Replication	We replicated ChIP-seq experiments for 4 transcription factors, performing the same experiment on different samples extracted on different days, using the same protocol. Similarly, all ATAC-seq experiments were carried out in duplicates, except for the control (rtTA3G) for which four replicates were performed.
Randomization	We used 100 randomly selected transcription factors to test the algorithm generated using machine learning. Those were picked randomly in the 501 quantified TFs, and we confirmed that the fraction of enriched transcription factors was the same in both training and testing data sets.
Blinding	This doesn't apply to our study.

Reporting for specific materials, systems and methods

Materials & experimental systems

n/a	Involved in the study	
☒	☐	Unique biological materials
☐	☒	Antibodies
☐	☒	Eukaryotic cell lines
☒	☐	Palaeontology
☒	☐	Animals and other organisms
☒	☐	Human research participants

Methods

n/a	Involved in the study	
☐	☒	ChIP-seq
☒	☐	Flow cytometry
☒	☐	MRI-based neuroimaging

Antibodies

Antibodies used	Anti-HA.11 IgG antibody BioLegend, clone 16B12, Cat# 901501 lots B231607, B201938, and B242906. Anti-Histone H3K9me3 antibody Abcam Cat# ab8898 lot GR275911-6.
Validation	https://www.biolegend.com/en-us/products/purified-anti-ha-11-epitope-tag-antibody-11374<br>https://www.abcam.com/histone-h3-tri-methyl-k9-antibody-chip-grade-ab8898.html

Eukaryotic cell lines

Policy information about cell lines

Cell line source(s)	E14: gift from Didier Trono; (EPFL)<br>CGR8: ATCC<br>NIH-3T3: provided by Ueli Schibler (University of Geneva)<br>HEK-293T cells: ATCC
Authentication	The E14 and NIH-3T3 cell lines were not authentified.
Mycoplasma contamination	E14 and NIH-3T3 cells were confirmed to be mycoplasma negative.
Commonly misidentified lines<br>(See ICLAC register)	HEK-293T: these cells were used for lentiviral vector production

ChIP-seq

Data deposition

☒ Confirm that both raw and final processed data have been deposited in a public database such as GEO.

☒ Confirm that you have deposited or provided access to graph files (e.g. BED files) for the called peaks.

Data access links<br>*May remain private before publication.*	GSE119784 (https://www.ncbi.nlm.nih.gov/geo/query/acc.cgi?acc=GSE119784, reviewer access token: "uxklyewcdbyjdir")
Files in database submission	SRA files for each replicate available in GEO. Additionally, processed files listed below:<br>GSM3383675_ATAC_3T3_WT.bw<br>GSM3383675_ATAC_3T3_WT_BAMPE_peaks.narrowPeak.gz<br>GSM3383706_BHLHB8.bw<br>GSM3383706_BHLHB8_filtered.bed.gz<br>GSM3383707_BHLHB8_2.bw<br>GSM3383707_BHLHB8_2_filtered.bed.gz<br>GSM3383708_BRACHYURY.bw<br>GSM3383708_BRACHYURY_filtered.bed.gz<br>GSM3383709_CDX2.bw<br>GSM3383709_CDX2_filtered.bed.gz<br>GSM3383710_DLX1.bw<br>GSM3383710_DLX1_filtered.bed.gz<br>GSM3383711_DLX6.bw<br>GSM3383711_DLX6_filtered.bed.gz<br>GSM3383712_DUXBL.bw<br>GSM3383712_DUXBL_filtered.bed.gz<br>GSM3383713_EBF1.bw<br>GSM3383713_EBF1_filtered.bed.gz<br>GSM3383714_FOXA1.bw<br>GSM3383714_FOXA1_filtered.bed.gz<br>GSM3383715_FOXA1_2.bw<br>GSM3383715_FOXA1_2_filtered.bed.gz<br>GSM3383716_FOXA1_RR.bw<br>GSM3383716_FOXA1_RR_filtered.bed.gz<br>GSM3383717_FOXA1_SW.bw<br>GSM3383717_FOXA1_SW_filtered.bed.gz<br>GSM3383718_HLF.bw<br>GSM3383718_HLF_filtered.bed.gz<br>GSM3383719_MAX.bw<br>GSM3383719_MAX_filtered.bed.gz<br>GSM3383720_NANOG.bw<br>GSM3383720_NANOG_filtered.bed.gz<br>GSM3383721_POU5F1.bw<br>GSM3383721_POU5F1_filtered.bed.gz<br>GSM3383722_POU5F1_2.bw<br>GSM3383722_POU5F1_2_filtered.bed.gz<br>GSM3383723_PRR3.bw<br>GSM3383723_PRR3_filtered.bed.gz<br>GSM3383724_RHOX11.bw<br>GSM3383724_RHOX11_filtered.bed.gz<br>GSM3383725_SIX6.bw<br>GSM3383725_SIX6_filtered.bed.gz<br>GSM3383726_SOX15.bw<br>GSM3383726_SOX15_filtered.bed.gz<br>GSM3383727_SOX2.bw

GSM3383727_SOX2_filtered.bed.gz
GSM3383728_SOX2_2.bw
GSM3383728_SOX2_2_filtered.bed.gz
GSM3383729_TEAD1.bw
GSM3383729_TEAD1_filtered.bed.gz
GSM3383730_THAP4.bw
GSM3383730_THAP4_filtered.bed.gz
GSM3383731_TOX3.bw
GSM3383731_TOX3_filtered.bed.gz
GSM3383732_ZFP319.bw
GSM3383732_ZFP319_filtered.bed.gz
GSM3383733_OCT4SOX2_3T3.bw
GSM3383733_OCT4SOX2_3T3_filtered.bed.gz
GSM3383734_Input.bw

Genome browser session
(e.g. UCSC)

http://genome-euro.ucsc.edu/cgi-bin/hgTracks?db=mm10&lastVirtModeType=default&lastVirtModeExtraState=&virtModeType=default&virtMode=0&nonVirtPosition=&position=chr19%3A1-61431566&hgsid=229112263_jey7513Ra2EexCkRQ0DyiwlLlvH0

Methodology

Replicates

ChIP-seq: One replicate per sample, except for BHLHB8, FOXA1, OCT4, and SOX2, for which two biological replicates were used. ATAC-seq: Two replicates per sample except for the control (rtTA3G), for which four replicates were used.

Sequencing depth

All sequencing experiments were paired end 2x37 bp. Find sequencing stats below.
Sample Percent_uniquely_mapped Reads_sequenced
ATAC_3T3_WT 79.61 70729740
ATAC_BHLHB8_1 72.29 41629556
ATAC_BHLHB8_2 72.89 43486495
ATAC_BRACHYURY_1 78.81 41595438
ATAC_BRACHYURY_2 70.17 46798948
ATAC_CDX2_1 75.63 62598907
ATAC_CDX2_2 77.1 35552350
ATAC_DLX6_1 72.09 35617981
ATAC_DLX6_2 73.49 41213171
ATAC_DUXBL_1 74.46 27887342
ATAC_DUXBL_2 77.83 42381189
ATAC_FOXA1_1 71.89 36966566
ATAC_FOXA1_2 73.16 40937165
ATAC_HLF_1 72.47 48515310
ATAC_HLF_2 71.29 43630428
ATAC_NANOG_1 71.48 49461276
ATAC_NANOG_2 71.6 37708170
ATAC_POU5F1_1 72.97 37526267
ATAC_POU5F1_2 73.79 44772082
ATAC_RHOX11_1 73.19 33719580
ATAC_RHOX11_2 72.44 40469280
ATAC_rtTA3G_1 70.76 29619317
ATAC_rtTA3G_2 73.06 41217656
ATAC_rtTA3G_3 72.68 46434873
ATAC_rtTA3G_4 70.85 45019358
ATAC_SIX6_1 74.88 38810515
ATAC_SIX6_2 70.09 46542507
ATAC_SOX15_1 76.39 23145498
ATAC_SOX15_2 75.19 24682706
ATAC_SOX2_1 72.74 32075913
ATAC_SOX2_2 78.4 31939738
ChIP_BHLHB8_1 79.97 67283687
ChIP_BHLHB8_2 81.91 29319297
ChIP_BRACHYURY 77.14 59301719
ChIP_CDX2 80.4 48158212
ChIP_DLX1 79.2 57813678
ChIP_DLX6 79.83 59073507
ChIP_DUXBL 79.19 27246231
ChIP_EBF1 78.76 46538811
ChIP_FOXA1_1 79.14 51312885
ChIP_FOXA1_2 78.74 36191428
ChIP_FOXA1_RR 79.67 31189726
ChIP_FOXA1_SW 79.29 33695200
ChIP_HLF 78.78 54953520
ChIP_Input 23.63 49609400
ChIP_MAX 80.36 28517596
ChIP_NANOG 78.35 54530643
ChIP_OCT4SOX2_3T3 75.76 27160167
ChIP_OCT4_1 79.21 29081679

ChIP_OCT4_2 78.69 31654536
ChIP_PRR3 79.31 29643879
ChIP_RHOX11 79.66 55581986
ChIP_SIX6 78.43 49916227
ChIP_SOX15 79.72 36653492
ChIP_SOX2_1 79.92 57384588
ChIP_SOX2_2 79.69 32736581
ChIP_SRCAP 79.03 32589106
ChIP_TEAD1 29.24 54722721
ChIP_THAP4 78.29 28490191
ChIP_TOX3 75.9 33175713
ChIP_ZFP319 79.12 28058818

Antibodies

Anti-HA.11 IgG antibody BioLegend, clone 16B12, Cat# 901501 lots B231607, B201938, and B242906.

Peak calling parameters

For each sample, peaks were called with MACS2 with settings '-f BAMPE -g mm'. Peaks overlapping peaks called for input (non-immunoprecipitated chromatin) from NIH-3T3 cells and ENCODE blacklisted peaks were discarded.

Data quality

All sequencing yielded 93-95 Q30% values. FastQC was used to check for good sequence quality (no samples were discarded). Duplicate reads were removed. All peaks called are FDR > 5% (q-value 0.05 in MACS2). Our correlations were quality-assured by (i) downsampling reads to the same number of reads, (ii) using a stringent q-value threshold (0.01), and (iii) using the alternative peak caller HOMER.

Software

STAR 2.5.3a, SAMTools 1.4, picard 2.8.3, HOMER 4.7, deepTools 2.4.2, BEDTools 2.2.6.0., MACS 2.1.1.20160309. R packages: GenomicRanges_1.28.6, edgeR_3.18.1, lima_3.32.10, ggplot2_3.0.0.
